# Interventions for cold homes: a rapid review of the health impacts

**DOI:** 10.1093/eurpub/ckae058

**Published:** 2024-04-08

**Authors:** Kimberly Lazo Green, Michelle M C Tan, Eugenie Evelynne Johnson, Nisar Ahmed, Claire Eastaugh, Fiona Beyer, Dawn Craig, Gemma F Spiers, Barbara Hanratty

**Affiliations:** Older People and Frailty Policy Research Unit, National Institute for Health and Care Research, The University of Manchester, Manchester, UK; Healthy Ageing Research Group, School of Health Sciences, Faculty of Biology, Medicine and Health, The University of Manchester, UK; National Institute for Health and Care Research Older People and Frailty Policy Research Unit, Newcastle University, Newcastle upon Tyne, UK; National Institute for Health and Care Research Older People and Frailty Policy Research Unit, Newcastle University, Newcastle upon Tyne, UK; Evidence Synthesis Group/Innovation Observatory, Population Health Sciences Institute, Newcastle University, Newcastle Upon Tyne, UK; Older People and Frailty Policy Research Unit, National Institute for Health and Care Research, The University of Manchester, Manchester, UK; Healthy Ageing Research Group, School of Health Sciences, Faculty of Biology, Medicine and Health, The University of Manchester, UK; National Institute for Health and Care Research Older People and Frailty Policy Research Unit, Newcastle University, Newcastle upon Tyne, UK; Evidence Synthesis Group/Innovation Observatory, Population Health Sciences Institute, Newcastle University, Newcastle Upon Tyne, UK; National Institute for Health and Care Research Older People and Frailty Policy Research Unit, Newcastle University, Newcastle upon Tyne, UK; Evidence Synthesis Group/Innovation Observatory, Population Health Sciences Institute, Newcastle University, Newcastle Upon Tyne, UK; National Institute for Health and Care Research Older People and Frailty Policy Research Unit, Newcastle University, Newcastle upon Tyne, UK; Evidence Synthesis Group/Innovation Observatory, Population Health Sciences Institute, Newcastle University, Newcastle Upon Tyne, UK; National Institute for Health and Care Research Older People and Frailty Policy Research Unit, Newcastle University, Newcastle upon Tyne, UK; Evidence Synthesis Group/Innovation Observatory, Population Health Sciences Institute, Newcastle University, Newcastle Upon Tyne, UK; National Institute for Health and Care Research Older People and Frailty Policy Research Unit, Newcastle University, Newcastle upon Tyne, UK

## Abstract

**Background:**

Cold homes are associated with an increased risk of adverse health outcomes for older people. To mitigate this risk, homes need to be heated to an appropriate temperature. This review aims to identify interventions designed to improve heating and temperatures within homes and summarize its impact on health, health service utilization and cost effectiveness.

**Methods:**

A rapid review was conducted. Studies assessing the effects of structural, financial, or behavioural interventions designed to improve home temperatures of residents aged 18+ years were eligible. Searches were carried out in four databases. A search for grey literature, and backward and forward citation searching were performed. Data were summarized in a narrative synthesis and mapped using EPPI-Reviewer and EPPI-Mapper software.

**Results:**

Eighteen studies reported across 19 publications were included. Structural interventions were associated with better mental health and quality of life, a reduction in health service utilization, and improvements in satisfaction with internal home temperature, social interactions and financial difficulties. The impact on physical health outcomes varied by age, gender and long-term conditions. Evidence about the impact of behavioural interventions was inconsistent.

**Conclusion:**

Structural improvements to increase home temperatures may offer the potential to improve some aspects of health. However, the impact on physical health, including which groups are most likely to benefit, is unclear. Key gaps include the lack of evidence about the impact of financial interventions, and the impact of all types of interventions, on quality of life, mortality and costs.

## Introduction

Cold homes are associated with health inequalities and increased risk of adverse health outcomes for older people.^1^ The United Kingdom (UK) has the sixth highest long-term rate of excess winter mortality out of 30 European countries, with an estimated 30% of excess deaths in England and Wales associated with living in homes with low temperatures in the winter period of 2021–2022.^2^^,^^3^ It is projected that in 2023, fuel poverty will increase to 14.4% (3.53 million) of households in England.^4^ Fuel poverty relates to a situation where a household needs to spend more than 10% of their adjusted net income in order to maintain a satisfactory warm indoor environment.^5^ Older people who are frail, socially isolated, at risk of falls, and/or have underlying health conditions like cardiovascular and respiratory problems, are likely to spend more of their time indoors.^6^ Therefore, older populations are especially vulnerable to the health consequences of cold homes, such as poor physical and mental health, and increased mortality risk.^7^

Action is needed to ensure homes are sufficiently heated. Preventable excess winter deaths can be reduced by improving the energy efficiency of homes and making heating systems more affordable.^8^ Local and national policies are focused on identifying approaches to reduce the health consequences of cold homes.^8^ Potential interventions include making structural changes to homes and heating systems (e.g. heating systems, insulation and double glazed windows),^9^^,^^10^ supplementing financial resources of older people to increase the affordability of heating (e.g. national fiscal or local funding schemes),^4^^,^^9^ and supporting behavioural changes around heating homes (e.g. using energy more efficiently).^11^

Although a number of systematic reviews have examined the overall effects of cold homes on health outcomes, mortality and morbidity, and well-being,^12^^,^^13^ only one has focused on identifying the types of interventions associated with health improvements.^10^ This review,^10^ published in 2013, found that structural improvements can lead to improved health outcomes especially for people with respiratory conditions. Additional evaluations of interventions to improve home temperatures have been published since this review, warranting an up-to-date and comprehensive synthesis of the evidence base. This study aimed to identify and summarize evidence on the effectiveness of interventions designed to improve heating and temperatures within homes to benefit health outcomes.

## Methods

We conducted a rapid review of primary studies to achieve a timely summary of evidence. Rapid reviews use modified systematic review methods to streamline study searches and selection, data extraction and quality assessment.^14^ The methods are reported following the Preferred Reporting Items for Systematic Reviews and Meta-Analyses (PRISMA) guidelines (Supplementary file S1).^15^

### Search strategy

Searches were carried out on 26th May 2023. The search strategy was developed by an information specialist in Applied Social Sciences Index and Abstracts on ProQuest and translated to other databases: MEDLINE (OVID), PsycINFO (OVID) and CINAHL (EBSCO). The concepts used were [population characteristics: cost of living] AND [population characteristics: housing] AND [interventions] and incorporated subject headings, synonyms, and text word searching. Searches were limited to publications dated from 1st January 2010 (Supplementary file S2). We anticipated that some evidence may be in non-peer reviewed sources; therefore, field experts were contacted to seek advice other non-peer reviewed publications that may be relevant. We also looked into the publication database of He Kainga Oranga Housing & Health. Grey literature was hand-searched based on these to supplement database searches. Backward and forward citation searching were carried out using Google Scholar.

### Eligibility criteria

Evaluations of structural, financial or behavioural interventions designed to improve home temperatures for the health benefit of residents aged 18 years or older were eligible (table 1). Eligible outcomes were any measure of physical or mental health, health service utilization, quality of life, mortality and cost effectiveness. Studies that reported non-health outcomes (e.g. mould, damp) were also included if they also reported health outcomes. Due to the changing nature of structural technologies for heating homes (e.g. heating systems, insulation etc.), we prioritized contemporary evidence published from 2010.

**Table 1:** Review criteria

**Population**	Adults (18+ years) living in residential homes (own homes, or rented accommodation, supported housing), with or without long-term health conditions. Populations living in group residential settings (e.g. halfway housing), and care homes with or without nursing are not eligible.
**Intervention**	Structural interventions that make changes to the infrastructure of homes for the purpose of improving home temperatures (e.g. changes to heating systems or insulation). Financial/fiscal interventions to support residents’ use of fuel or structural changes to homes (e.g. income supplementation targeted at fuel poverty relief, local funding schemes, national fiscal schemes). Behavioural interventions to change the way people use energy within their homes (e.g. information and advice about heating homes). Interventions delivered for any period are eligible.
**Comparator**	Any comparator or none (i.e. before and after studies).
**Outcome**	Primary outcomes Any measure of physical or mental health; any measure of health service utilization or impact on health systems (e.g. hospital waiting times); mortality; quality of life, and cost effectiveness. Secondary outcomes Non-health outcomes such as levels of damp and mould are eligible if reported alongside health outcomes.
**Study design**	Randomized and non-randomized trials, non-controlled before and after studies. Studies published in English from 2010.

### Study screening and selection

Titles and abstracts were screened independently by two reviewers (E.E.J., N.S.). Disagreements were resolved through discussion or arbitrated by a third reviewer (G.S.). The full texts of selected records were retrieved and assessed independently by two reviewers using a hierarchy of exclusion criteria (K.G., M.T.) (Supplementary file S3). All records were managed in Rayyan,^16^ an online platform to support systematic review screening.

### Data extraction and quality assessment

A data extraction form was developed and piloted. Two reviewers independently performed the data extraction (K.G., M.T.). Fifty percent of extracted data were checked by another researcher (G.S.). Studies were critically appraised using the NIH Quality Assessment Tool for Randomised studies, and Controlled and Before-and-After Studies.^17^ An overall rating of ‘poor’ was given if two or more of the criteria were unmet; ‘fair’ if one criterion was unmet and ‘good’ if all criteria were met.

### Synthesis

Evidence was summarized using a narrative synthesis. We grouped studies by intervention category (i.e. structural, behavioural and financial) and then outcome (i.e. physical health, mental health, quality of life, health service utilization, mortality, cost effectiveness and non-health outcomes). We reported the effectiveness of the interventions by examining the overall direction of effects across studies. Narrative subgroup analysis was conducted on studies that reported outcomes by age (60+ or all adult ages), populations with long-term conditions [e.g. Chronic obstructive pulmonary disease (COPD), heart disease] or socioeconomic status.

An evidence map was produced to visualize the volume and concentration of evidence by intervention type and outcome. A single researcher (E.E.J.) coded records of included studies in EPPI-Reviewer software.^18^ The coding report was downloaded from EPPI-Reviewer and checked for accuracy against the data extraction by a second researcher (K.G.). The evidence map was then generated using the EPPI-Mapper wizard.

## Results

**Figure 1:**
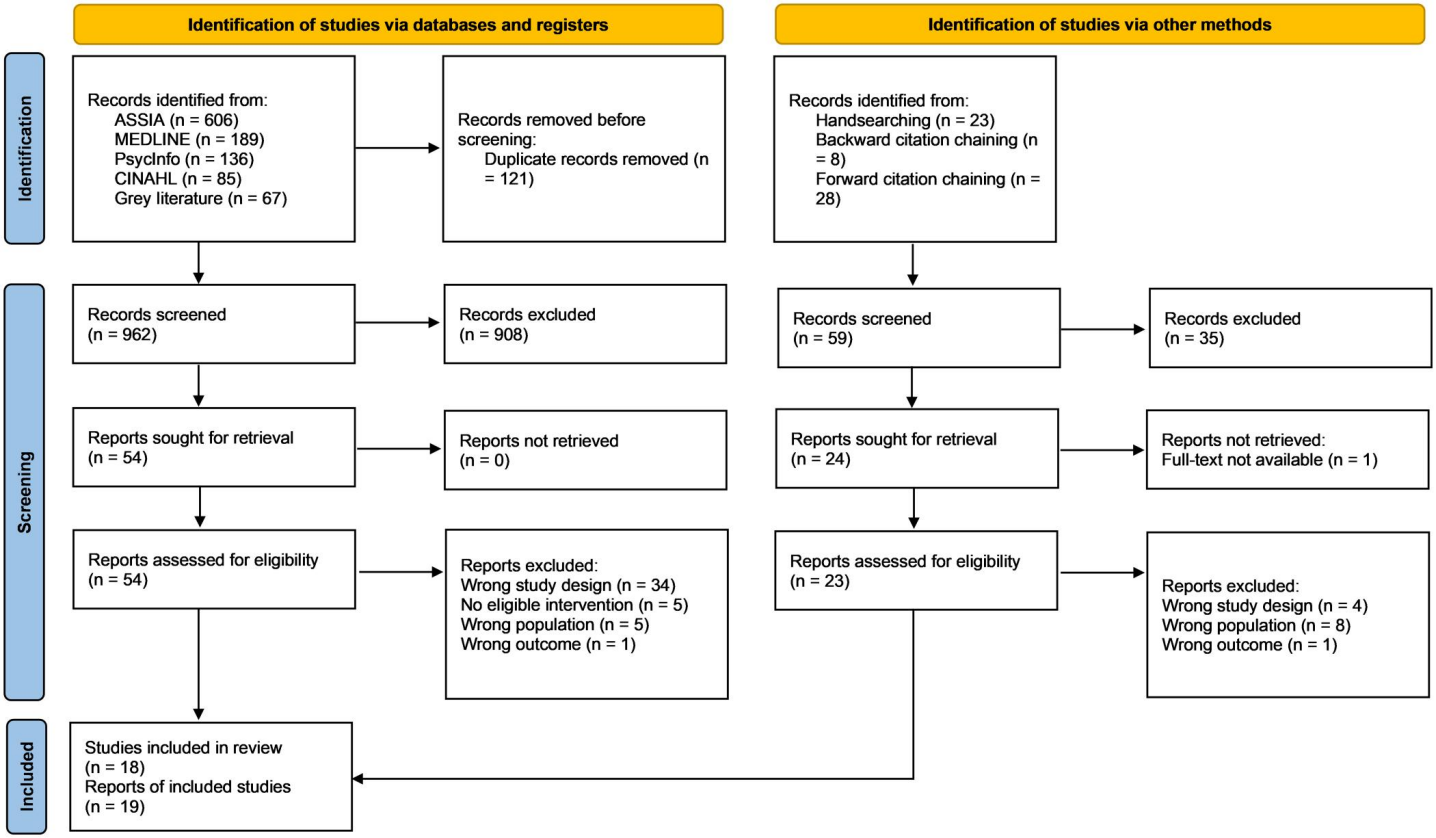
Prisma diagram. *Source*: Page MJ, McKenzie JE, Bossuyt PM, Boutron I, Hoffmann TC, Mulrow CD, et al. The PRISMA 2020 statement: an updated guideline for reporting systematic reviews. BMJ 2021;372:n71. doi: 10.1136/bmj.n71.

After screening, 18 studies reported across 19 publications met the criteria and were included in this review (figure 1). Three studies were reported across six publications (two publications per study)^19–24^ and three studies^25^ were reported in one publication.

### Study characteristics

Four studies used randomized designs^28–31^ and 14 used non-randomized designs,^19–25^^,^^32–39^ including prospective cohort (*n* = 5), retrospective (*n* = 6) and before and after (*n* = 3). Five non-randomized studies reported cost analyses.^19^^,^^22^^,^^24^^,^^25^^,^^30^^,^^32^ Overall, half of the included studies specifically reported evidence about older adults aged 60 years and over.^20^^,^^21^^,^^23^^,^^24^^,^^29–31^^,^^34^^,^^37–39^ Five studies reported evidence for populations aged 18 years and over^19^^,^^22^^,^^28^^,^^32^^,^^33^^,^^34^ and a minority of studies reported evidence for all ages (0–60+), where we extracted outcomes for studies of population aged 18 years and over.^25^^,^^36^ Some studies specifically looked at adults living in social or affordable housing,^19^^,^^21^^,^^22^^,^^25^^,^^36^ hard-to-treat homes,^25^ and/or those experiencing fuel poverty.^28^^,^^32^^,^^35^ Eleven studies included populations with long-term health conditions: respiratory (e.g. asthma and COPD) and/or cardiovascular (e.g. high blood pressure) diseases.^20^^,^^23–25^^,^^29^^,^^34–39^ A summary of all studies included in this review is presented in table 2.

**Table 2:** Summary of study characteristics

Author, year	Study design	Country	Age group	Range or mean (SD) age	Sample size	Target population (Condition)	Type of intervention	Quality rating
Carrere et. al. 2022^32^	Non-RCT (Quasi-experiment with control, Prospective)	Spain	18 and over	Year of birth, SD Intervention: 1965 (15.9) Control: 1969 (15.9)	Total *n* = 394 Intervention *n* = 246 Control *n* = 148	People who are energy poor, defined as those who are unable to ensure socially and materially required levels of domestic energy services	Behavioural	Poor
Pollard et. al. 2019^35^	Non-RCT (Cross-sectional, Prospective)	UK	18 and over	The majority of participants completing the questionnaire (73%) were aged over 65 years of age.	*n* = 22 households	Fuel poor, households that struggle with the costs of keeping their home warm due to the cost of fuel and energy efficiency of the property	Behavioural	Poor
Saeki et. al. 2015^31^	RCT (Prospective)	Japan	60 and over	71.6 (6.6) years	*n* = 359	Aged 60 and over living at home	Behavioural	Fair
Bray et. al. 2017^19^	Non-RCT (Historical cohort and cost analysis, Prospective)	UK	25 and over	25–34: 15 (6.6%) 35–44: 23 (10.1%) 44–54: 36 (15.8%) 55–65: 43 (18.9%) 65–74: 44 (19.3%) 75+: 66 (29.9%)	228 households	Social housing	Structural	Poor
Edwards et. al. 2016^22^	Non-RCT (Historical cohort, Prospective)	UK	25 and over	25–34: 15 (6.6%), 35–44: 23 (10.1%) 44–54: 36 (15.8%) 55–65: 43 (18.9%) 65–74: 44 (19.3%) 75+: 66 (29.9%)	228 households	Families living in social housing in Sunderland.	Structural	Fair
Curl and Kearns 2015^20^	Non-RCT (Longitudinal study, Retrospective)	UK	65 and over	Control, no improvements (*n* = 602): 27.8% Intervention groups: Central heating (*n* = 374) 22.2% Kitchen and bathroom (*n* = 706) 29.5% Doors (*n* = 483) 26.5% Fabric works (*n* = 575) 31.3% Whole sample (*n* = 1993) 28.3%	Wave 1, 2006 *n* = 6003 Wave 2, 2008 *n* = 4869 Wave 3, 2011 *n* = 4270 Time 1 sample matched Wave 1 and 2 *n* = 1933 Time 2 sample matched Wave 2 and 3 *n* = 1933	Respiratory and circulatory diseases	Structural	Fair
Curl et. al. 2015^21^	Non-RCT (Longitudinal study, Retrospective)	UK	65 and over	Control, no improvements (*n* = 602): 27.8% Intervention groups: Central heating (*n* = 374) 22.2% Kitchen and bathroom (*n* = 706) 29.5% Doors (*n* = 483) 26.5% Fabric works (*n* = 575) 31.3% Whole sample (*n* = 1993) 28.3%	Wave 1, 2006 *n* = 6003 Wave 2, 2008 *n* = 4869 Wave 3, 2011 *n* = 4270 Time 1 sample matched Wave 1 and 2 *n* = 1933 Time 2 sample matched Wave 2 and 3 *n* = 1934	Social housing	Structural	Fair
Fyfe 2021^23^	Non-RCT (Quasi-experimental cohort study, Retrospective)	New Zealand	All ages, extracted outcomes for 65 and over	Intervention: 0–4 *n* = 66 634 (13.44%) 11–14 *n* = 66 392 (13.39%) 15–24 *n* = 68 947 (13.9%) 25–34 *n* = 76 106 (15.35%) 35–44 *n* = 71 114 (15.35%) 45–54 *n* = 52 237 (10.53%) 55–64 *n* = 40 911 (8.25%) 75–84 *n* = 17 756 (3.58%) 85+ *n* = 5342 (0.88%) Control: 0–4 *n* = 58 649 (11.52%) 11–14 *n* = 72 815 (14.31%) 15–24 *n* = 79 335 (15.59%) 25–34 *n* = 76 605 (15.05%) 35–44 *n* = 70 157 (13.79%) 45–54 *n* = 55 994 (11%) 55–64 *n* = 42 495 (8.35%) 75–84 *n* = 17 843 (3.51%) 85+ *n* = 5413 (1.06%)	*n* = 235 772 houses Intervention *n* = 495 864 Control *n* = 508 931 Study population: *n* = 1 004 825 Deaths during the study: *n* = 16 337	Residents of owner-occupied and private rental dwellings	Structural	Fair
Telfar-Barnard et. al. 2011^24^	Non-RCT (Cohort study and cost analysis, Retrospective)	New Zealand	All ages, extracted outcome for 65 and over	Adults aged 19–64: 63 121 (58.76%) Older people (65+): 18 103 (16.85%)	Total treatment cohort at that time = 107 421		Structural	Fair
Poortinga et. al. 2018^25^	Non-RCT (Cohort analysis, Retrospective)	UK	All ages	NA	*n* = 25 908 individuals living in 4968 intervention	Social housing and COPD, cardiovascular conditions	Structural	Fair
Poortinga et. al. 2018^25^^,^^26^^,^^27^	Non-RCT (Quasi-experimental before and after study, Prospective)	UK	All ages	≤ 25 years: 1.6% (14/776) 26–35: 6.4% (50/776) 36–45: 10.1% (78/776) 46–54: 12.6% (98/776) 55–64: 27.3% (213/776) ≥ 65: 41.6% (323/776)	*n* = 352 intervention *n* = 418 control	Hard-to-heat, hard-to-treat Social housing and COPD, cardiovascular conditions	Structural	Poor
Poortinga et. al. 2018^25^^,^^26^^,^^27^	Non-RCT (Cost analysis, Prospective)	UK	All ages	≤ 25 years: 1.6% (14/776) 26–35: 6.4% (50/776) 36–45: 10.1% (78/776) 46–54: 12.6% (98/776) 55–64: 27.3% (213/776) ≥ 65: 41.6% (323/776)	NA	Social housing	Structural	Fair
Heyman et al. 2011^28^	RCT (Prospective)	UK	20 and over	NA	237 households	Households in full or marginal fuel poverty	Structural	Poor
Liddell et. al. 2011^33^	Non-RCT (Cohort and cost analysis, Prospective)	UK	25 and over	Age 25–44: Intervention: 32 493 Control: 265 619 Age 45–59: Intervention: 17 823 Control: 162 098 Age 60+: Intervention: 22 523 Control: 135 916	165 686 households	Houses in the private sector	Structural	Poor
Osman et. al. 2010^29^	RCT (Prospective)	UK	65 and over	All: 69.6 (8.5) Intervention: 71 (10) Control: 68 (7.2) Monitor: 69 (7.9)	*n* = 178	Clinician-diagnosed COPD	Structural	Fair
Page et. al. 2022^30^	RCT (Cost analysis, Prospective)	Australia	60 and over	74.8 (11.5)	*n* = 1312 individuals, 984 households 2018: *n* = 143 268 households 2019: *n* = 356 268 households 2020: *n* = 813 611 households Control: *n* = 493 Intervention: *n* = =te	Most participants had either never smoked (52%) or had given up >12 months ago (39%); only 3% were current smokers	Structural	Poor
Peralta et. al. 2017^34^	Non-RCT (Time-stratified case-crossover analysis, Retrospective)	Spain	60 and over	Men: No intervention: 69.18 (12.81) Intervention: 72.65 (13.88) Women: No intervention: 75.52 (13.67) Intervention: 78.68 (12.16)	*n* = 2252	Deaths of people living in the studied blocks and occurred in the city during the cold periods.	Structural	Poor
Poortinga et. al. 2017^36^	Non-RCT (Cross-sectional study, Five-wave repeated, Prospective)	UK	All ages	Age under 36: 15% (year 2009), 15% (2011), 15% (2012), 15% (2014), 12% (2016). Age 36–45: 12% (year 2009), 11% (2011), 10% (2012), 10% (2014), 10% (2016). Age 46–54: 11% (year 2009), 11% (2011), 12% (2012), 11% (2014), 12% (2016). Age 55–64: 17 (year 2009), 16 (2011), 17 (2012), 16 (2014), 16 (2016). Age 65+: 41 (year 2009), 44 (2011), 45 (2012), 48 (2014), 48 (2016).	*n* = 10 009	Social housing	Structural	Fair
Preval et. al. 2017^37^	Non-RCT (Quasi-experimental cohort study, Retrospective)	New Zealand	60 and over	Circulatory Insulation: 78.8 (6.9) Heating: 78.3 (7.2) Insulation and heating: 78.9 (7), Control: 78.5 (7.2) Respiratory Insulation: 78.2 (7.3) Heating: 78.3 (5.9) Insulation and heating: 79.2 (8.0) Control: 77.5 (7.2)	*n* = 4848	Cardiovascular and respiratory-related hospitalization	Structural	Fair
Rodgers et. al. 2018^38^	Non-RCT (Longitudinal study, Retrospective)	UK	All ages, extracted outcome for 60 and over	Intervention: <25 *n* = 13 943 (43.6%) 25–39 *n* = 5435 (17%) 40–49 *n* = 2922 (9.1%) 50–59 *n* = 2655 (8.3%) 60–69 *n* = 2774 (8.7%) 70–79 *n* = 2362 (7.4%) 80+ *n* = 1918 (6%) Control: <25 *n* = 81 899 (35.4%) 25–39 *n* = 43 885 (19%) 40–49 *n* = 29 393 (12.7%) 50–59 *n* = 28 681 (12.4%) 60–69 *n* = 22 767 (9.8%) 70–79 *n* = 14 895 (6.4%) 80+ *n* = 9680 (4.2%)	Intervention *n* = 32 009 Control *n* = 231 200	Tenants aged 60 and over, cardiovascular, respiratory and injuries	Structural	Poor
Tonn et. al. 2023^39^	Non-RCT (Quasi-experimental)	USA	60 and over	Mean 64 Treatment: 60 Control: 60	382 multifamily buildings *n* = 1921 households *n* = 2964 persons	Affordable multifamily building offered by property owners that had 5+ units.	Structural	Fair

COPD, clinician-diagnosed chronic obstructive pulmonary disease; NA, not applicable; RCT, randomized controlled trial; SD, standard deviation; UK, United Kingdom.

Green: RCTs, Orange: non-RCTs; Blue: linked studies.

Fifteen studies assessed structural interventions^19–30^^,^^33^^,^^34^^,^^36–39^ and three assessed behavioural interventions.^31^^,^^32^^,^^35^ No studies assessing the health impact of financial interventions were found. The duration of interventions ranged from 3 months to 20 years. The most common components of structural interventions consisted of the installation of insulation retrofits (*n* = 12), heating improvements (*n* = 11) and installation of new double-glazed windows to replace single-glazed windows (*n* = 8). For the three behavioural interventions, the components were home visits for energy counselling, instructions to change thermostat settings, and the use of wearable telemetry to measure blood pressure. Characteristics of the intervention studies are presented in table 3.

**Table 3:** Features of interventions

Author, year	Study design	Type of intervention	Name of intervention and stakeholder involvement	Funding amount	Components of intervention	Duration of intervention (assessment points)
Carrere et. al. 2022^32^	Non-RCT	Behavioural	Energia, la Justa (EJUSTA): Partly funded by a research grant from the Carlos III Institute of Health, Ministry of Economy and Competitiveness (Spain) co-funded with European Union ERDF funds (European Regional Development Fund)	NA	Energy-counselling home visits to: (i) protect people’s energy via legal action, (ii) promote energy-saving behaviours to achieve savings, (iii) optimization of energy services through change of provide or tariffs, and (iv) installation of micro efficiency measures, that is, plug-in timers, energy saving light bulb, etc.	5 months, 2016 (Baseline and 1 year after intervention)
Pollard et al. 2019^35^	Non-RCT	Behavioural	Wearable telemetry: Funded by The British Gas Energy Trust Healthy Homes Fund 2015	NA	Wearable telemetry (i.e. a thermometer with a low-temperature alarm) The loggers were equipped with sensors that triggered a flashing light-emitting diode (LED) if the temperature in the immediate vicinity fell below 18°C or rose above 26°C. This provided participants and their families with an alert for changes in temperature that could exacerbate their LSI. Temperatures were recorded every 15 minutes with the number of readings below 18°C and 15°C noted.	3 months, winter 2016/2017 (Baseline and after three months)
Saeki et al. 2015^31^	RCT	Behavioural	Instruction in home heating on indoor temperature: Department of Indoor Environmental Medicine, Nara Medical University; JSPS KAKENHI; Mitsui Sumitomo Insurance Welfare Foundation; Meiji Yasuda Life Foundation of Health and Welfare; Osaka Gas Group Welfare Foundation; Japan Diabetes Foundation; Daiwa Securities Health Foundation; and the Japan Science and Technology Agency	NA	Participants were asked to set the heating device in the living room to start 1 hour before the estimated rising time with a target temperature of 24°C, and to stay in the living room until 2 hours after rising as long as possible	1 year, December–March 2010, September–March 2012 (Baseline and after intervention)
Bray et. al. 2017^19^	Non-RCT	Structural	The Warm Homes for Health: Funded by Gentoo and Nottingham City Homes	Mean total cost of the intervention per household was £3725.26 (SD=£1041.48)	The intervention consisted of the installation of (i) new double-glazed windows to replace single-glazed windows, and (ii) installation of a new energy-efficient combi boiler.	8 months, April–December 2014 (Baseline and 1 year after intervention)
Edwards et. al. 2016^22^	Non-RCT	Structural		The average cost of improving each house was £3725. This comprised a new combi boiler (£2500 per boiler) and double-glazing (£240 per window)		
Curl and Kearns 2015^20^	Non-RCT	Structural	GoWell project: Funded by the Scottish government	NA	(i) Central Heating, (ii) Front Doors, (iii) Windows, (iv) Environmental, (v) Fabric Works, (vi) Internal Common Works, (vii) Lift Replacement and (viii) Kitchen, Bathroom & Rewiring.	2 years, 2006 (Wave 1)–2008 (Wave 2)
Curl et. al. 2015^21^	Non-RCT	Structural		£1.2 billion		3 years, 2006–2011 [Time 1 (before) and Time 2 (after) Three ‘wave pairings; Wave 1 - Wave 2, Wave 2 - Wave 3, and Wave 1 - Wave 3, which are recoded into Time 1 and Time 2]
Fyfe 2021^23^	Non-RCT	Structural	Warm Up New Zealand: Funded by the New Zealand government, delivered by Energy Efficiency and Conservation Authority (EECA)	NZ$347 million	Insulation retrofits and clean, efficient heating grants for New Zealand households	5 years, 2009 to 2014 (2 years)
Telfar-Barnard et al 2011^24^	Non-RCT	Structural		NA		4 years, 2009–2013 (Baseline and after intervention, 1 January 2008–30 September 2010.)
Poortinga et al. 2018^25^	Non-RCT	Structural	Welsh Government Warm Homes programme: Funded by Welsh Government	£68 million	The programme funded retrofit measures such as (i) solid wall insulation, (ii) solar panels, (iii) solar hot water and (iv) heat pumps. Additional options were available such as (v) boiler upgrades and replacements, (vi) window upgrades, (vii) roof extensions, (viii) structural work and (ix) energy saving advice	1 year, 2010–2011 (Baseline and after intervention)
Poortinga et al. 2018^25^	Non-RCT	Structural		£45 million		3 years, 2012–2015 (Baseline and 2 years from baseline)
Poortinga et al. 2018^25^	Non-RCT	Structural		£68 million		5 years, 2010–2015 (Phase 1: 2010–2011; Phase 2: 2012–2015)
Heyman et. al. 2011^28^	RCT	Structural	Generic energy efficiency intervention: Funded by the UK National Lottery Community Fund. Partner organizations in the delivery of the intervention National Energy Action, North Tyneside Council, Northern Electric	Intervention group households received an individually tailored package of improved heating and insulation. Control group households received heating and insulation package. The packages were worth an average of £727 (range £0–3335), and included loft insulation (54%), cavity wall insulation (53%), draught exclusion (29%), heating controls (20%), central heating (13%) and other measures as required	Tailored package of improved (i) heating and (ii) insulation	4 years, 2000–2004 (End of year 1 and end of year 4)
Liddell et. al. 2011^33^	Non-RCT	Structural	Kirklees Warm Zone Project: Funded by Kirklees council and Scottish Power. Central government contributed through Warm Front scheme. Co-delivered with Kirklees Energy Services, Scottish Power, National Grid, Citizens’ Advice, the Pensions Service, Kirklees Benefits Advice Service and Revenue and Benefits, Warm Zone Ltd., Miller Pattison, West Yorkshire Fire Service, Carers Gateway, Yorkshire Water, Commissioned assessors	£24 million	(i) Free cavity wall insulation, (ii) free low energy light bulbs, (iii) free improvements to heating systems, (iv) competitive prices for replacement boilers and central heating and (v) interest free loans for renewable technologies	3 years, 2007–2010 (After intervention)
Osman et. al. 2010^29^	RCT	Structural	Affordable Warmth Scheme: Co-delivered with Castlehill Housing Association (Care and Repair)	NA	Improvements included replacement and upgrades to (i) central heating systems, installation of (ii) loft, under-floor and cavity wall insulation and (iii) benefit reassessment.	18 months, 2004–2007 (Baseline and 1 year after intervention)
Page et. al. 2022^30^	RCT	Structural	Victorian Healthy Homes Program (VHHP): Initial program funding was granted by the Sustainability Fund of the Victorian Government and additional funding was provided by Sustainability Victoria (SV)	The program allowed for an average of $3,500 per home to be spent on labour and materials to improve thermal comfort in an energy-efficient way. This was reduced to a target average of $2600 in the final year of the program	(i) Draught sealing (ii) Insulation—new or top-up ceiling, underfloor (iii) Space heating and cooling (iv) Window Furnishings (v) Lighting	3 years, 2018–2020 (3-month winter period of their study year)
Peralta et al. 2017^34^	Non-RCT	Structural	Energy efficiency facade retrofitting (EEFR): Funded by European Community’s Seventh Framework Programme	NA	Types of EEFR not reported	20 years, 1982–2012 [Day of death (Lag 0) to 20th day before death (Lag 20)]
Poortinga et al. 2017^36^	Non-RCT	Structural	Carmarthenshire Homes Standard: Funded by Carmathernshire County Council	£200+ million	The programme involved the elements of (i) windows and doors; (ii) boilers; (iii) kitchens; (iv) bathrooms; (v) electrics; (vi) loft insulation; (vii) cavity-wall insulation; (viii) external wall insulation and (ix) safety improvements to external paths.	7 years, 2009–2016 (2009, 2011, 2012, 2014, 2016)
Preval et al 2017^37^	Non-RCT	Structural	Warm Up New Zealand: Heat Smart Programme: Funded by New Zealand government, delivered by Energy Efficiency and Conservation Authority (EECA)	$347 million	Installation of insulation retrofits and clean, efficient heating grants	4 years, 2009–2013 (2 years)
Rodgers et al 2018^38^	Non-RCT	Structural	Housing co-interventions: Council	NA	The eight cointerventions were new (i) windows and doors, (ii) kitchens, (iii) bathrooms, (iv) heating systems, (v) wall insulation, (vi) loft insulation, (vii) electrical systems and (viii) garden paths	10 years dataset, January 2005 and March 2015 (10 years)
Tonn et al 2023^39^	Non-RCT	Structural	Weatherization: One sponsor of this work is based in New York City and the other set of sponsors were utility companies in the Commonwealth of Massachusetts.	NA	Buildings assigned to the Comparison with Treatment or Treatment groups had or were expected to have installed major weatherization measures (i.e. air sealing, insulation, HVAC repair and replacement). Phase 1 and 2 surveys were carried out.	2 years, 2018–2020 (Baseline and after intervention)

NZ, New Zealand; RCT, randomized controlled trial; SD, standard deviation.

Green, RCTs, Orange, non-RCTs; Blue, linked studies.

### Critical appraisal results

Two of the four randomized controlled trials (RCTs) were rated poor quality,^28^^,^^30^ and two were fair.^29^^,^^31^ The main sources of bias in the RCTs were the large dropout rates and the lack of information to determine blinding of participants and treatment allocation. For the non-randomized studies, seven were rated fair^20–22^^,^^24^^,^^25^^,^^36^^,^^37^ and eight were rated poor quality.^19^^,^^25^^,^^32–35^^,^^38^^,^^39^ The main sources of bias in the non-randomized studies were the lack of blinding of outcome assessors to the participants’ interventions and poor consistency in outcome measures across multiple time points before and after the interventions (Supplementary file S4).

### Effectiveness of interventions

Summary data on effectiveness for each intervention are detailed in Supplementary file S5.

#### Behavioural interventions

Three studies evaluated behavioural interventions.^31^^,^^32^^,^^35^ Outcomes evaluated in these studies included: physical health, mental health, quality of life, health service utilization, household temperature and fuel poverty. Two of these studies were rated as poor quality and one study was rated as fair.

Physical health outcomes were assessed in two studies^31^^,^^32^ using self-reported health measures, systolic and diastolic blood pressure surges, and minutes spent in physical activity. These studies produced conflicting evidence. One showed no improvements,^32^ while the other illustrated positive improvements^31^ in physical health as a result of the behavioural interventions.

Two studies^32^^,^^35^ reported mental health outcomes using self-reported measures on depression and/or anxiety, the use of antidepressants or sleeping medication, and feeling of vulnerability, cold, or illness during the winter period. Interventions had no significant impact on depression and/or anxiety^32^ and on self-reported measures of feeling cold or poorly during winter, compared to the previous year.^35^ However, participants felt less vulnerable after the intervention, all of whom were more aware of the temperature inside their homes after wearing telemetry.^35^ In contrast, there was a modest but statistically insignificant increase in antidepressant or sleeping medication use in the intervention group.^32^

One study^35^ reported changes in quality of life using self-reported well-being measures. The behavioural intervention had no significant effect on the overall well-being of the participants.

In terms of health service utilization, two studies^32^^,^^35^ measured the frequency of primary care visits per year, and the use of other National Health Service (NHS) services, including Accident and Emergency department (A&E) and pharmacy for any respiratory, cardiovascular and/or cold/flu symptoms. The impact of behavioural interventions on primary care visits was inconsistent. One study^32^ showed a significant decrease in primary care visits as a result of the intervention. The other study^35^ showed a significant increase in the frequency of primary care service utilization, which was linked to decreased use of A&E and pharmacist services.

Two studies^31^^,^^32^ assessed non-health outcomes, including indoor temperature, self-reported measures of keeping appropriate temperature during winter months, and arrears on utility bills. Both studies^31^^,^^32^ reported significant increase in indoor temperatures, although one study used a subjective measure to assess this (participants’ awareness of ambient temperatures).^32^ There was also a significant decrease in utility bill arrears in one study,^32^ although the reduction was larger for the non-intervention group.

#### Structural interventions

Fifteen studies evaluated structural interventions.^19–30^^,^^33^^,^^34^^,^^36–39^ Outcomes in these studies included: mortality, physical health, mental health, quality of life, health service utilization, cost effectiveness and a number of non-health outcomes. The quality rating of these studies were poor for eight studies and fair for eight.

Mortality was assessed in three studies^23^^,^^24^^,^^30^^,^^34^ using all-cause and cold-associated mortality rates. Varied effects were observed across all studies by age groups, gender and types of long-term diseases. In three studies, all-cause mortality for adults aged 60 and over did not improve following a structural intervention.^23^^,^^30^^,^^34^ However, only one study adjusted for sociodemographic factors.^23^ One study, which was rated low in quality, reported that the intervention acted as a risk factor,^34^ increasing the cold-associated deaths particularly for older men, as well as older adults with respiratory and circulatory diseases. In another study, there was a lower cumulative cold-associated mortality for older adults aged 65 and over who received insulation and heating compared with other types of structural interventions.^23^ In terms of deaths with an all-cause or cold-associated hospital admission, there was no significant effect for adults aged 65 and over.^23^^,^^24^ In contrast, a significant decrease in mortality was reported for older adults who were hospitalized with circulatory diseases.^24^

Physical health was assessed in five studies.^20^^,^^25^^,^^28^^,^^36^^,^^39^ One study used objective measures, such as time spent on moderate or vigorous physical activities,^39^ while the remaining studies recorded self-reported symptoms^25^^,^^28^^,^^36^ and association of interventions in preventing or recovering from self-reported respiratory or circulatory symptoms.^20^ Symptoms for respiratory and cardiovascular diseases were recorded by five studies.^20^^,^^25^^,^^28^^,^^36^^,^^39^

There was inconsistent evidence about the impact of structural interventions on improving these symptoms. One study showed improvement in asthma symptoms (e.g. wheezing) and chronic headaches following structural upgrades.^39^ Central heating was effective in preventing the development of circulatory and respiratory diseases in the long-term.^20^ Cavity wall insulation, windows and doors and boilers had no significant impact in improving symptoms of respiratory and circulatory diseases.^36^ An increase in physical activity was found for households aged 60 and over, albeit this improvement was statistically insignificant.^39^

Four studies^20^^,^^25^^,^^36^^,^^39^ assessed mental health outcomes. This was measured using subjective well-being scales, self-reported stress and/or depression scores, and association of interventions in preventing mental health diseases. Two studies^20^^,^^36^ suggested an improvement in mental health conditions, such as long-term stress, anxiety or depression. Installing cavity wall insulation was associated with better mental health for some households living in social housing,^36^ while central heating was reported to be effective in preventing mental health diseases for adults over the age of 65.^20^

Six studies^19^^,^^21^^,^^22^^,^^25^^,^^28–30^ recorded health-related quality of life outcomes using a variety of scales. Most of the studies reported improvements.^19^^,^^21^^,^^22^^,^^28^^,^^30^ Housing improvements, such as doors, fabric works and kitchen and bathrooms, as well as central heating, were all associated with a positive change in mental well-being components for older adults aged 65 and over.^21^

Health service utilization was assessed by nine studies^19^^,^^22^^,^^23^^,^^25^^,^^28–30^^,^^37–39^ using measures such as hospital, A&E, and primary care visits, rate of prescriptions dispensed and emergency and routine hospital admission rates. Most of the evidence suggested an overall significant reduction in health service utilization after implementing structural interventions. Results varied per age group and type of long-term condition. Overall general practitioner visits, routine hospital visits, and A&E attendance significantly reduced after structural improvements^19^^,^^22^^,^^25^^,^^39^ for households with respiratory conditions and those in social/affordable housing. However, one study reported a statistically significant increase in doctor visits for symptoms related to shortness of breath, bronchitis or other COPD, or emphysema flare ups.^39^ In three studies, hospital admission rates fell only for older adults aged 60 and over who have cardiovascular diseases, such as COPD and/or heart conditions.^23^^,^^25^^,^^37^ No significant change in hospital admissions was found for other populations.^39^ Structural interventions that were associated with fewer hospital admissions were insulation,^23^^,^^37^ heating and electrical systems.^38^

Five studies^19^^,^^22^^,^^24^^,^^25^^,^^30^^,^^33^ reported cost analyses of structural interventions. Evidence across the five studies suggested that the interventions produced overall cost savings and reduced health services expenditure. Estimates of total savings on health services ranged from £2000 to £150 000^30^^,^^33^ and £1.1 to £10 million value on lives saved.^25^^,^^33^ This was primarily associated with a reduction in use of health services and lower mortality rates.

Non-health outcomes, such as room temperatures, home warmth satisfaction and thermal comfort, social interactions, and financial difficulties, were reported by six studies.^19^^,^^22^^,^^25^^,^^28–30^^,^^36^ Structural housing improvements were associated with modest increase in indoor room temperatures in one study.^28^ This was not the case for households over 65 years and over diagnosed with COPD in another study,^29^ where no change in temperatures was found. Structural interventions were also associated with satisfaction with internal home temperature,^25^^,^^28^^,^^30^^,^^36^ increased social interactions,^25^ and reduced severity of financial difficulties.^25^ Two studies reported no impact on humidity^29^ and mould.^30^

### Evidence map

The evidence map (https://eppi.ioe.ac.uk/cms/Portals/35/Maps/NIHRPRU/O1_%20ColdHomeInterventions.html) highlights the concentration of evidence by types of intervention and outcomes, segmented by study design and with a filter for age range (reporting data for people under or over 60 years). The highest concentration of evidence was found for structural interventions investigating physical health, mental health and health service utilization outcomes. Key evidence gaps were observed for mortality and costs outcomes, as well as financial and behavioural interventions.

## Discussion

This rapid review found that structural interventions (e.g. heating systems, insulation, double glazed windows) are promising to improve mental health outcomes, quality of life and some aspects of physical health. The impact on mortality and physical health outcomes was inconsistent. Where improvements in physical health were reported for structural interventions, these effects tended to be weaker for older populations, particularly older people with respiratory and cardiovascular conditions.

Structural interventions were linked to health improvements, similar to the findings of previous studies.^10^ Cavity wall insulation and central heating were associated with better mental health,^20^^,^^36^ central heating was effective in preventing circulatory and respiratory symptoms,^20^ and electrical systems were associated with reduced hospital admissions.^38^ Structural interventions may also reduce certain types of health service use, with potential for cost savings. Only one study reported that insulation and heaters were linked to reduced mortality, but only for those aged 65 who had previously been hospitalized with circulatory illness.^24^ Across some of these studies reporting mortality, there was a lack of information on adjustment for important confounders, warranting caution in the interpretation of the findings.

A very small evidence base about behavioural interventions did not demonstrate consistent health benefits. This may be due to differences in the interventions, study populations and outcome measures across the three evaluations. Notably, two of the behavioural interventions were delivered to populations considered to be ‘energy-poor’^32^^,^^35^. In both studies, the authors suggested that the ability of energy-poor households to heat their home and/or change heating behaviours, largely depend on other factors, such as household income, state of the property (e.g. lack of ventilation etc.) and/or the residents’ health risks.

A key evidence gap identified in this review is around evaluations of financial interventions to improve home temperatures for health benefits. This may reflect the paucity of such interventions, rather than an absence of evaluation. One such intervention does exist in the UK: The Winter Fuel Payment. However, we found no evaluations of the impact of this scheme on health outcomes. A study published in 2019 assessed eligibility for the Winter Fuel Payment: no clear health improvements were observed. This study was ineligible for our review as it considered populations who were eligible for, rather than populations who received, this supplementation.^40^

A final observation is that the overall quality of the identified evidence was weak, with around half of studies judged to be poor. Thus, while the evidence points to the potential benefits of structural interventions to improve some health outcomes, this finding should be interpreted with caution.

### Limitations

Our approach in this review has enabled us to produce a comprehensive synthesis of evidence about approaches to tackling poor health resulting from cold homes. Extensive searches across peer-reviewed and grey literature ensured this synthesis was exhaustive; our inclusion of behavioural, financial and structural interventions guaranteed it was comprehensive. A limitation of our approach is that we excluded evidence published before 2010. This was to ensure our synthesis was not undermined by evidence of outdated structural technologies. Consequently, we may have missed evidence about other types of interventions published before this date. However, we do not believe this to be a major risk as our preliminary scoping did not identify evaluations of non-structural interventions prior to this period.

### Implications for practice and future research

Mitigating the health impact of cold homes is a policy priority. Evidence suggests that structural interventions are promising to improve mental health, quality of life and reduce some health care utilization. However, the impact on physical health is less clear, and unlikely to be uniform across populations. Further evidence about the impact of structural (and other) interventions on mortality is also needed.

## Conclusions

Structural improvements to homes to increase home temperatures are promising to improve some aspects of health, quality of life and reduce utilization of some types of healthcare. It may also offer cost savings from reduced health service use. Further evidence is needed to understand the impact on mortality. Behavioural interventions did not demonstrate consistent benefits to physical or mental health, or to health service utilization. Key gaps include evaluations of the impact of financial interventions on health, and the impact of all interventions on mortality, quality of life and cost effectiveness outcomes.

## Supplementary Material

ckae058_Supplementary_Data

## Data Availability

The data that support the findings of this study are available from the corresponding author upon reasonable request.

## Abbreviations

A&E: Accident and Emergency department
COPD: Chronic obstructive pulmonary disease
NHS: National Health Service
RCT: Randomized controlled trial
SES: Socioeconomic status
UK: United Kingdom
