# Supplementary material for: Interventions for cold homes: a rapid review of the health impacts
Source: Eur J Public Health. 2024 Apr 8;34(4):682–95. doi: 10.1093/eurpub/ckae058 (PMC11293810; doi:10.1093/eurpub/ckae058)
Supplement: ckae058_Supplementary_Data [file ckae058_supplementary_data.pdf]

Supplementary Files

Supplementary File 1. PRISMA checklist

Section and Topic	Item #	Checklist item	Location where item is reported
**TITLE**			
Title	1	Identify the report as a systematic review.	Page 1
**ABSTRACT**			
Abstract	2	See the PRISMA 2020 for Abstracts checklist.	Page 2
**INTRODUCTION**			
Rationale	3	Describe the rationale for the review in the context of existing knowledge.	Page 3
Objectives	4	Provide an explicit statement of the objective(s) or question(s) the review addresses.	Page 3
**METHODS**			
Eligibility criteria	5	Specify the inclusion and exclusion criteria for the review and how studies were grouped for the syntheses.	Page 4 and Table 1
Information sources	6	Specify all databases, registers, websites, organisations, reference lists and other sources searched or consulted to identify studies. Specify the date when each source was last searched or consulted.	Page 4
Search strategy	7	Present the full search strategies for all databases, registers and websites, including any filters and limits used.	Supplementary File 2
Selection process	8	Specify the methods used to decide whether a study met the inclusion criteria of the review, including how many reviewers screened each record and each report retrieved, whether they worked independently, and if applicable, details of automation tools used in the process.	Pages 4 to 5
Data collection process	9	Specify the methods used to collect data from reports, including how many reviewers collected data from each report, whether they worked independently, any processes for obtaining or confirming data from study investigators, and if applicable, details of automation tools used in the process.	Page 5
Data items	10a	List and define all outcomes for which data were sought. Specify whether all results that were compatible with each outcome domain in each study were sought (e.g. for all measures, time points, analyses), and if not, the methods used to decide which results to collect.	Page 5
	10b	List and define all other variables for which data were sought (e.g. participant and intervention characteristics, funding sources). Describe any assumptions made about any missing or unclear information.	Page 5 and Table 2
Study risk of bias assessment	11	Specify the methods used to assess risk of bias in the included studies, including details of the tool(s) used, how many reviewers assessed each study and whether they worked independently, and if applicable, details of automation tools used in the process.	Page 5
Effect measures	12	Specify for each outcome the effect measure(s) (e.g. risk ratio, mean difference) used in the synthesis or presentation of results.	NA
Synthesis methods	13a	Describe the processes used to decide which studies were eligible for each synthesis (e.g. tabulating the study intervention characteristics and comparing against the planned groups for each synthesis (item #5)).	Page 5

Section and Topic	Item #	Checklist item	Location where item is reported
	13b	Describe any methods required to prepare the data for presentation or synthesis, such as handling of missing summary statistics, or data conversions.	Page 5
	13c	Describe any methods used to tabulate or visually display results of individual studies and syntheses.	Page 5
	13d	Describe any methods used to synthesize results and provide a rationale for the choice(s). If meta-analysis was performed, describe the model(s), method(s) to identify the presence and extent of statistical heterogeneity, and software package(s) used.	Page 5
	13e	Describe any methods used to explore possible causes of heterogeneity among study results (e.g. subgroup analysis, meta-regression).	NA
	13f	Describe any sensitivity analyses conducted to assess robustness of the synthesized results.	NA
Reporting bias assessment	14	Describe any methods used to assess risk of bias due to missing results in a synthesis (arising from reporting biases).	NA
Certainty assessment	15	Describe any methods used to assess certainty (or confidence) in the body of evidence for an outcome.	NA
**RESULTS**			
Study selection	16a	Describe the results of the search and selection process, from the number of records identified in the search to the number of studies included in the review, ideally using a flow diagram.	Page 6 and Figure 1
	16b	Cite studies that might appear to meet the inclusion criteria, but which were excluded, and explain why they were excluded.	NA
Study characteristics	17	Cite each included study and present its characteristics.	Page 6
Risk of bias in studies	18	Present assessments of risk of bias for each included study.	Page 6
Results of individual studies	19	For all outcomes, present, for each study: (a) summary statistics for each group (where appropriate) and (b) an effect estimate and its precision (e.g. confidence/credible interval), ideally using structured tables or plots.	Pages 7 to 10
Results of syntheses	20a	For each synthesis, briefly summarise the characteristics and risk of bias among contributing studies.	Pages 7 to 10
	20b	Present results of all statistical syntheses conducted. If meta-analysis was done, present for each the summary estimate and its precision (e.g. confidence/credible interval) and measures of statistical heterogeneity. If comparing groups, describe the direction of the effect.	NA
	20c	Present results of all investigations of possible causes of heterogeneity among study results.	NA
	20d	Present results of all sensitivity analyses conducted to assess the robustness of the synthesized results.	NA
Reporting biases	21	Present assessments of risk of bias due to missing results (arising from reporting biases) for each synthesis assessed.	NA
Certainty of evidence	22	Present assessments of certainty (or confidence) in the body of evidence for each outcome assessed.	NA
**DISCUSSION**			
Discussion	23a	Provide a general interpretation of the results in the context of other evidence.	Pages 10 to 11

Section and Topic	Item #	Checklist item	Location where item is reported
	23b	Discuss any limitations of the evidence included in the review.	Page 12
	23c	Discuss any limitations of the review processes used.	Page 12
	23d	Discuss implications of the results for practice, policy, and future research.	Page 12
**OTHER INFORMATION**			
Registration and protocol	24a	Provide registration information for the review, including register name and registration number, or state that the review was not registered.	NA
	24b	Indicate where the review protocol can be accessed, or state that a protocol was not prepared.	NA
	24c	Describe and explain any amendments to information provided at registration or in the protocol.	NA
Support	25	Describe sources of financial or non-financial support for the review, and the role of the funders or sponsors in the review.	Page 13
Competing interests	26	Declare any competing interests of review authors.	Page 14
Availability of data, code and other materials	27	Report which of the following are publicly available and where they can be found: template data collection forms; data extracted from included studies; data used for all analyses; analytic code; any other materials used in the review.	Page 16

From: Page MJ, McKenzie JE, Bossuyt PM, Boutron I, Hoffmann TC, Mulrow CD, et al. The PRISMA 2020 statement: an updated guideline for reporting systematic reviews. BMJ 2021;372:n71. doi: 10.1136/bmj.n71

For more information, visit: http://www.prisma-statement.org/

Supplementary File 2. Search Strategy

Searches carried out 26/05/23 by CE, Research Assistant Information Specialist, Evidence Synthesis Group.

Summary of results

**Database**	**Search date**	**Results**
ASSIA	26/05/23	603
Medline	26/05/23	189
PsycINFO	26/05/23	136
CINHAL	26/05/23	85
**Total BEFORE de-duplication**		1013
**Total AFTER de-duplication**		797

**Database**	**Search date**	**Results**
ASSIA	13/06/23*	606
Medline	26/05/23	189
PsycINFO	26/05/23	136
CINHAL	26/05/23	85
**Total BEFORE de-duplication**		1016
**Total AFTER de-duplication**		895

*Searches carried out 26/05/2023 across all databases. ASSIA search re-ran 13/06/23 due to an export error. Following the re-run, 3 additional records were retrieved. After deduplication, 98 additional records were sent for screening.

Search strategy as applied to ASSIA. Search date: 26/05/23

#	Searches	Results
1	TITLE((temperature NEAR/3 home*) OR (warm* NEAR/3 home*) OR (afford* NEAR/3 warm*) OR (keep* warm*) OR (cold* NEAR/3 home*) OR (freez* NEAR/3 home*) OR "fuel poverty" OR "fuel poor" OR "living standard*" OR "cost of living" OR "inadequate warmth" OR "vulnerable household*") OR ABSTRACT((temperature NEAR/3 home*) OR (warm* NEAR/3 home*) OR (afford* NEAR/3 warm*) OR (keep* warm*) OR (cold* NEAR/3 home*) OR (freez* NEAR/3 home*) OR "fuel poverty" OR "fuel poor" OR "living standard*" OR "cost of living" OR "inadequate warmth" OR "vulnerable household*") OR MAINSUBJECT.EXACT.EXPLODE("Poverty") OR MAINSUBJECT.EXACT("Cost of living")	11,095
2	TITLE(home OR house OR houses OR housing OR domicile* OR occupan* OR tenant OR tenancy) OR ABSTRACT(home OR house OR houses OR housing OR domicile* OR occupan* OR tenant OR tenancy) OR MAINSUBJECT.EXACT.EXPLODE("Housing") OR MAINSUBJECT.EXACT("Households")	77,055
3	TITLE(energy OR consumption OR heating OR insulation OR "energy efficiency" OR "switch* supplier" OR "switch* tariff" OR "warmth improvement" OR welfare OR benefit* OR grant* OR scheme* OR intervention* OR (recei* NEAR/2 benefit*)) OR ABSTRACT(energy OR consumption OR heating OR insulation OR "energy efficiency" OR "switch* supplier" OR "switch* tariff" OR "warmth improvement" OR welfare OR benefit* OR grant* OR scheme* OR intervention* OR (recei* NEAR/2 benefit*)) OR MAINSUBJECT.EXACT("Heating") OR MAINSUBJECT.EXACT("Energy consumption") OR MAINSUBJECT.EXACT.EXPLODE("Financial support") OR	248,889

	MAINSUBJECT.EXACT("Welfare benefits") OR MAINSUBJECT.EXACT("Social welfare") OR MAINSUBJECT.EXACT("Social security")	
4	1 AND 2 AND 3	806
5	pd(20100101-20231231)	603

Search date: 26/05/2023

#	Searches	Results
1	TITLE((temperature NEAR/3 home*) OR (warm* NEAR/3 home*) OR (afford* NEAR/3 warm*) OR (keep* warm*) OR (cold* NEAR/3 home*) OR (freez* NEAR/3 home*) OR "fuel poverty" OR "fuel poor" OR "living standard*" OR "cost of living" OR "inadequate warmth" OR "vulnerable household*") OR ABSTRACT((temperature NEAR/3 home*) OR (warm* NEAR/3 home*) OR (afford* NEAR/3 warm*) OR (keep* warm*) OR (cold* NEAR/3 home*) OR (freez* NEAR/3 home*) OR "fuel poverty" OR "fuel poor" OR "living standard*" OR "cost of living" OR "inadequate warmth" OR "vulnerable household*") OR MAINSUBJECT.EXACT.EXPLODE("Poverty") OR MAINSUBJECT.EXACT("Cost of living")	11,138
2	TITLE(home OR house OR houses OR housing OR domicile* OR occupan* OR tenant OR tenancy) OR ABSTRACT(home OR house OR houses OR housing OR domicile* OR occupan* OR tenant OR tenancy) OR MAINSUBJECT.EXACT.EXPLODE("Housing") OR MAINSUBJECT.EXACT("Households")	77,221
3	TITLE(energy OR consumption OR heating OR insulation OR "energy efficiency" OR "switch* supplier" OR "switch* tariff" OR "warmth improvement" OR welfare OR benefit* OR grant* OR scheme* OR intervention* OR (recei* NEAR/2 benefit*)) OR ABSTRACT(energy OR consumption OR heating OR insulation OR "energy efficiency" OR "switch* supplier" OR "switch* tariff" OR "warmth improvement" OR welfare OR benefit* OR grant* OR scheme* OR intervention* OR (recei* NEAR/2 benefit*)) OR MAINSUBJECT.EXACT("Heating") OR MAINSUBJECT.EXACT("Energy consumption") OR	249,742

	MAINSUBJECT.EXACT.EXPLODE("Financial support") OR MAINSUBJECT.EXACT("Welfare benefits") OR MAINSUBJECT.EXACT("Social welfare") OR MAINSUBJECT.EXACT("Social security")	
4	1 AND 2 AND 3	809
5	pd(20100101-20231231)	606

Grey literature search

Date accessed	URL
19/04/2023	https://assets.publishing.service.gov.uk/government/uploads/system/uploads/attachment_data/file/355790/Briefing7_Fuel_poverty_health_inequalities.pdf
19/04/2023	https://www.nhsglos.nhs.uk/news/warm-home-scheme-supports-vulnerable-people-to-pay-their-energy-bills/
19/04/2023	https://www.kirklees.gov.uk/beta/delivering-services/pdf/warmzone-process-report.pdf
19/04/2023	https://www.gov.uk/the-warm-home-discount-scheme
19/04/2023	https://www.changeworks.org.uk/case-studies/external-wall-insulation-transforms-borders-home
19/04/2023	https://localenergy.scot/case-studies/?pg=3
19/04/2023	https://www.healthyhousing.org.nz/our-research/past-research/evaluation-warm-new-zealand-heat-smart-programme
19/04/2023	https://www.wakefield.gov.uk/Documents/housing/wakefield-heca-report-2021.pdf
19/04/2023	https://khub.net/documents/135939561/174099487/Cold+Weather+Plan+-+Making+the+Case.pdf/69c22c83-1b1f-cfb9-0e2d-8f603a7b426e?t=1635339855693
19/04/2023	https://www.ofgem.gov.uk/environmental-and-social-schemes/energy-company-obligation-eco
19/04/2023	https://www.ageuk.org.uk/our-impact/programmes/safe-and-warm/
19/04/2023	https://cafs.org.uk/cold-to-cosy-homes-eligibility-criteria/
19/04/2023	https://www.cornwall.gov.uk/council-news/communities-and-housing/energy-saving-improvements-made-to-more-than-600-cornwall-council-homes/
19/04/2023	https://www.trustmark.org.uk/news/press-releases/2022/11/16/home-energy-efficiency-experts-suppliers-gather-to-provide-retrofit-guidance

19/04/2023	https://www.nice.org.uk/sharedlearning/wigan-council-s-affordable-warmth-access-referral-mechanism-awarm---the-original-single-point-of-contact-health-and-housing-referral-service-for-people-living-in-cold-homes-as-recommended-by-nice-guidelines-ng6
19/04/2023	https://www.nea.org.uk/
19/04/2023	https://www.nea.org.uk/who-we-are/innovation-technical-evaluation/domestic-batteries-devon/
04/05/2023	https://link.springer.com/article/10.1186/s12889-022-12994-4
04/05/2023	https://www.instituteofhealthequity.org/resources-reports/fuel-poverty-cold-homes-and-health-inequalities-in-the-uk/read-the-report.pdf
04/05/2023	https://onlinelibrary.wiley.com/doi/full/10.1111/ina.13101
09/05/2023	https://www.ageuk.org.uk/globalassets/age-uk/documents/reports-and-publications/reports-and-briefings/safe-at-home/age-uk-energy-public-policy-report-march-2023.pdf
09/05/2023	https://www.nea.org.uk/wp-content/uploads/2023/01/3830_NEA_Fuel-Poverty-Monitor-Report-2022_V2-1.pdf
09/05/2023	https://ageing-better.org.uk/sites/default/files/2022-03/CaCHE-Past-Present-and-Future-Housing-Policy.pdf
09/05/2023	https://www.housinglin.org.uk/_assets/Resources/Housing/Support_materials/Practice_briefings/HLIN_PracticeBriefing_HealthInequalities.pdf
09/05/2023	https://www.housinglin.org.uk/Topics/type/Cold-Homes-Toolkit/
09/05/2023	https://www.housinglin.org.uk/Topics/type/The-cost-of-cold-why-we-need-to-protect-the-health-of-older-people-in-winter/
09/05/2023	https://www.housinglin.org.uk/Topics/type/Warm-homes-for-health-briefing/
09/05/2023	https://www.housinglin.org.uk/Topics/type/Building-comfort-for-old-age-Designing-and-managing-thermal-comfort-in-low-carbon-housing-for-older-people/
09/05/2023	https://www.nice.org.uk/guidance/ng6
10/05/2023	https://www.britishgas.co.uk/energy/energy-saving/warm-home-discount.html
10/05/2023	https://www.britishgas.co.uk/home-services/home-improvements/powerflush.html

10/05/2023	https://www.britishgas.co.uk/smart-home.html#products
10/05/2023	https://www.britishgas.co.uk/smart-home/smart-meters.html
10/05/2023	https://www.britishgas.co.uk/home-services/boilers-and-heating/air-source-heat-pumps.html
10/05/2023	https://www.britishgas.co.uk/smart-home/greener-homes/solar-products.html
10/05/2023	https://www.britishgas.co.uk/energy/smart-export-guarantee.html
10/05/2023	https://www.edfenergy.com/help-support/warm-home-discount-0
10/05/2023	https://www.edfenergy.com/heating/insulation
10/05/2023	https://www.edfenergy.com/heating/insulation/loft
10/05/2023	https://www.edfenergy.com/heating/insulation/cavity-wall
10/05/2023	https://www.edfenergy.com/solar
10/05/2023	https://www.edfenergy.com/energy-efficiency/smart-export-tariff
10/05/2023	https://www.edfenergy.com/eco4-subsidised-energy-efficiency-measures
10/05/2023	https://www.edfenergy.com/energy-efficiency/the-great-british-insulation-scheme
10/05/2023	https://www.edfenergy.com/smart-meters
10/05/2023	https://www.eonnext.com/warm-home-discount
10/05/2023	https://www.eonnext.com/policies/energy-efficiency
10/05/2023	https://www.eonnext.com/smart
10/05/2023	https://www.scottishpower.co.uk/ebss
10/05/2023	https://www.scottishpower.co.uk/ebss/alternative-fuel-payment

10/05/2023	https://www.scottishpower.co.uk/solar-panels
10/05/2023	https://web-content.scottishpower.co.uk/files/pdf/Better_Home_Cooler%20Planet_Final_Web_Report_19_July_2022.pdf?nocache=true
10/05/2023	https://www.scottishpower.co.uk/air-source-heat-pumps
10/05/2023	https://www.scottishpower.co.uk/energy-efficiency/smart-meters
10/05/2023	https://www.scottishpower.co.uk/tado
10/05/2023	https://www.sse.com/what-we-do/net-zero-acceleration-programme/
10/05/2023	https://www.ssen-transmission.co.uk/projects/2030-projects/
10/05/2023	https://www.ovoenergy.com/guides/energy-guides/warm-home-discount-scheme
10/05/2023	https://www.ovoenergy.com/smart-meters
10/05/2023	https://www.ovoenergy.com/smart-home/smart-thermostat
10/05/2023	https://www.ovoenergy.com/heat-pumps
10/05/2023	https://www.ovoenergy.com/solar-panels
10/05/2023	https://www.ovoenergy.com/energy-tracker
10/05/2023	https://assets.publishing.service.gov.uk/government/uploads/system/uploads/attachment_data/file/355790/Briefing7_Fuel_poverty_health_inequalities.pdf
10/05/2023	https://beatcold.org.uk/wp-content/uploads/2011/09/Awarm-evaluation-final-report.pdf
10/05/2023	https://assets.publishing.service.gov.uk/government/uploads/system/uploads/attachment_data/file/451025/DECC_FINAL.pdf

Supplementary File 3. Hierarchy of exclusion criteria

1. Is the study published in the English language? If yes, go to Q2. If no, exclude on LANGUAGE.
2. Is the study published from 2010 onwards? If yes, go to Q3. If no, exclude on DATE.
3. Does the study report an evaluation (quantitative or mixed methods)? If yes, go to Q4. If no, exclude on STUDY DESIGN.
4. Does the study evaluate an eligible intervention? If yes, go to Q5. If no, exclude on INTERVENTION.
5. Does the study evaluate the intervention for populations aged 18+? If yes, go to Q6. If no, exclude on POPULATION.
6. Does the study evaluate eligible outcomes? If yes, INCLUDE. If no, exclude on OUTCOME.

Supplementary File 4: Quality appraisal of included studies using NIH Quality Assessment Tool (RCTs)

**Author (year)**	***Osman et al 2010***	***Heyman et al 2011***	***Saeki et al 2015***	***Page et al 2022***
Was the study described as randomized, a randomized trial, a randomized clinical trial, or an RCT?	Yes	Yes	Yes	Yes
Was the method of randomization adequate (i.e., use of randomly generated assignment)?	Not reported	Yes	Yes	Yes
Was the treatment allocation concealed (so that assignments could not be predicted)?	Not reported	Cannot determine	Yes	Cannot determine
Were study participants and providers blinded to treatment group assignment?	Not reported	Not reported	Not reported	Cannot determine
Were the people assessing the outcomes blinded to the participants' group assignments?	Not reported	Not reported	Not reported	Cannot determine
Were the groups similar at baseline on important characteristics that could affect outcomes (e.g., demographics, risk factors, co-morbid conditions)?	Yes	Yes	Yes	Yes
Was the overall drop-out rate from the study at endpoint 20% or lower of the number allocated to treatment?	Yes	No	Yes	No
Was the differential drop-out rate (between treatment groups) at endpoint 15 percentage points or lower?	Yes	No	Not reported	No
Was there high adherence to the intervention protocols for each treatment group?	Yes	Not applicable	Yes	Yes
Were other interventions avoided or similar in the groups (e.g., similar background treatments)?	Not reported	No	Not reported	Not reported
Were outcomes assessed using valid and reliable measures, implemented consistently across all study participants?	Yes	Yes	Yes	Yes
Did the authors report that the sample size was sufficiently large to be able to detect a difference in the main outcome between groups with at least 80% power?	Yes	Not reported	Not reported	Not reported
Were outcomes reported or subgroups analyzed prespecified (i.e., identified before analyses were conducted)?	Yes	Yes	Yes	Yes

Were all randomized participants analyzed in the group to which they were originally assigned, i.e., did they use an intention-to-treat analysis?	Yes	No	No	Yes
Overall Quality Rating (Good, Fair, or Poor)	**Fair**	**Poor**	**Fair**	**Poor**

Quality appraisal of included studies using NIH Quality Assessment Tool (non-RCTs)

Author (year)	***Liddell et al 2011***	***Telfar-Barnard et al 2011***	***Curl and Kearns 2015***	***Curl et al 2015***	***Edwards et al 2016***	***Bray et al 2017***	***Poortinga et al 2017***	***Preval et al 2017***	***Peralta et al 2017***	***Poortinga et al 2018a***	***Poortinga et al 2018b***	***Poortinga et al 2018c***	***Rodgers et al 2018***	***Pollard et al 2019***	***Fyfe 2021***	***Carrere et al 2022***	***Tonn et al 2023***
Was the study question or objective clearly stated?	Yes	Yes	Yes	Yes	Yes	Yes	Yes	Yes	Yes	Yes	Yes	Yes	Yes	Cannot determine	Yes	Yes	Yes
Were eligibility/selection criteria for the study population prespecified and clearly described?	Yes	Yes	Yes	Yes	Yes	Yes	Yes	Yes	Not applicable	Yes	Yes	Yes	Yes	No	Yes	Yes	No
Were the participants in the study representative of those who would be eligible for the test/service/intervention in the general or clinical population of interest?	Yes	Yes	Yes	Yes	Not reported	Yes	Yes	Yes	Cannot determine	Yes	Yes	Yes	Yes	No	Yes	Yes	Cannot determine
Were all eligible participants that met the prespecified entry criteria enrolled?	Yes	Yes	Yes	Yes	Yes	Yes	Yes	Yes	Not applicable	Yes	Yes	Cannot determine	Yes	Yes	Yes	Cannot determine	Cannot determine

Was the sample size sufficiently large to provide confidence in the findings?	Yes	Yes	Yes	Yes	Cannot determine	Yes	Yes	Yes	Cannot determine	Yes	Yes	Yes	Yes	No	Yes	Yes	Yes
Was the test/service/intervention clearly described and delivered consistently across the study population?	Yes	Yes	Yes	Yes	Yes	Yes	Yes	Yes	Not applicable	Yes	Yes	Yes	Yes	Yes	Yes	Yes	Yes
Were the outcome measures prespecified, clearly defined, valid, reliable, and assessed consistently across all study participants?	Yes	Yes	No	No	Yes	Yes	Yes	No	Yes	Yes	Yes	Yes	No	Yes	Yes	Yes	Yes
Were the people assessing the outcomes blinded to the participants' exposures/interventions?	No	Not applicable	Not applicable	Not applicable	No	No	Not reported	Not reported	No	Not applicable	Not applicable	Not applicable	Not applicable	No	Not reported	No	Not applicable
Was the loss to follow-up after baseline 20% or less? Were those lost to follow-up accounted for in the analysis?	Not reported	Not applicable	Not applicable	Not applicable	Not reported	No	Not reported	Not applicable	Not reported	Not reported	No	Not reported	Not applicable	Not reported	Not applicable	No	Not applicable
Did the statistical methods examine changes in outcome measures from before to	Not reported	Yes	Yes	Yes	No	Yes	No	Yes	Not applicable	Yes	Yes	Not applicable	Yes	No	Yes	Yes	Yes

after the intervention? Were statistical tests done that provided p values for the pre-to-post changes?																	
Were outcome measures of interest taken multiple times before the intervention and multiple times after the intervention (i.e., did they use an interrupted time-series design)?	No	No	Yes	Yes	No	No	Not applica ble	Yes	No	No	No	No	No	No	No	No	No
If the intervention was conducted at a group level (e.g., a whole hospital, a community, etc.) did the statistical analysis take into account the use of individual-level data to determine effects at the group level?	Yes	Yes	Yes	Yes	No	No	Not applica ble	Yes	No	Yes	Yes	Cannot determi ne	Yes	No	Yes	No	No
Overall Quality Rating (Good, Fair, or Poor)	**Poor**	**Fair**	**Fair**	**Fair**	**Fair**	**Po or**	**Fair**	**Fair**	**Poor**	**Fair**	**Poor**	**Fair**	**Poor**	**Poor**	**Fair**	**Poor**	**Poor**

Supplementary File 5. Effectiveness of interventions: data summary table

Author, year	Type of intervention	Outcomes	Outcome measures	Key statistical findings
Bray et al 2017	Structural	Quality of life	Self-rated household health status, as measured using a visual analogue scale (VAS)	t = -2.652, p=0.009
			Self-rated main tenant health status, as measured using a visual analogue scale (VAS)	t = -3.564, p<0.001
			Main tenant health-related quality-of-life score, EQ-5D-3L validated health-related quality-of-life (HRQoL)	t = 0.583
			Main tenant well-being score, Short Warwick-Edinburgh Well-being Scale (SWEMWBS)	t = -1.403
			Main tenantLife satisfaction score, ONS personal well-being measure	t = -0.615
			Main tenant happiness score, ONS personal well-being measure	t = -1.573
			Main tenant anxiety score, ONS personal well-being measure	t = 1.518
			Main tenant financial satisfaction score, ONS personal well-being measure	t = -2.340, p=0.020
		Non-health outcomes	Rooms left unheated, fuel poverty	t = 5.973; p<0.001
		Cost analysis	General practitioner, Health service use costs on primary care and secondary care, frequency	MC = -0.65 (9.07); Cost change -£24.94 (-£94.87, £30.63)
			Hospital inpatient, Health service use costs on primary care and secondary care, frequency	MC = -0.01 (0.88); Cost change -£9.34 (-£133.09, £116.74)
			Hospital outpient, Health service use costs on primary care and secondary care, frequency	MC = -0.30 (1.31), p=0.001; Cost change -£38.86 (-£64.57, -£15.43)
			Accident/emergency, Health service use costs on primary care and secondary care, frequency	MC = -0.18 (1.06); Cost change -£30.99 (-£57.33, -£3.10)
			Total, Health service use costs on primary care and secondary care, frequency	-£94.79 (-£273.01, £85.14)

Carrere et. al. 2022	Behavioural	Physical health<br>Mental health<br>Health service utilisation<br>Non-health outcomes	Self-perceived poor health<br>Depression and/or anxiety<br>Use of anxiolytics, antidepressant or sleeping pills<br>Primary care visits a year<br>Inability to keep home adequately cool<br>Inability to keep home adequately warm<br>Arrears utility bills	% Ave ME: − 0.9% (-13.2%, 11.4%)<br>% Ave ME: 1.3% (-18.6%, 21.2%)<br>% Ave ME: 8.6% (-0.1%, 17.3%)<br>% Ave ME: − 27.3% (-42.0%, -12.6%), p<0.001<br>% Ave ME: − 27.3% (-46.4%, -8.2%), p<0.01<br>% Ave ME: − 30.4% (-55.0%, -5.8%), p<0.01<br>% Ave ME: − 21.5% (2.3%, 40.7%), p<0.05
Curl and Kearns 2015	Structural	Physical health	Recovery from respiratory conditions, central heating<br>Recovery from circulatory conditions, central heating<br>Prevention of respiratory diseases, central heating<br>Prevention of circulatory diseases, central heating<br>Prevention of respiratory diseases if remain in employment, central heating<br>Prevention of circulatory diseases if remain in employment, central heating<br>Prevention of respiratory diseases if w/ pre-existing conditions, central heating<br>Prevention of circulatory diseases if w/ pre-existing conditions, central heating<br>Prevention of respiratory diseases in 5 years, central heating<br>Prevention of respiratory diseases in 2 years, central heating<br>Prevention of circulatory diseases in 5 years, central heating<br>Prevention of circulatory diseases in 2 years, central heating<br>Prevention of mental health diseases, central heating	OR = 0.81 (0.35, 1.9)<br>OR = 2.63 (1.17, 5.92), p<0.05<br>OR = 1.25 (0.82, 1.91)<br>OR = 1.02 (0.69, 1.53)<br>OR = 0.35 (0.16, 0.76), p<0.01<br>OR = 0.16 (0.06, 0.37), p<0.01<br>OR = 2.05 (1.48, 2.83), p<0.01<br>OR = 1.07 (0.77, 1.5)<br>OR = 2.79 (1.72, 4.52), p<0.01<br>OR = 1.83 (1.03, 3.25), p<0.05<br>OR = 2.39 (1.54, 3.74), p<0.01<br>OR = 1.71 (1.02, 2.87), p<0.05<br>OR = 1.01 (0.26, 3.97)

		Mental health	Prevention of mental health diseases if remain in employment, central heating	OR = 0.17 (0.03, 0.83), p<0.05
			Prevention of mental health diseases if over 65, central heating	OR = 0.25 (0.06, 0.98), p<0.001
Curl et al 2015	Structural	Quality of life	Association of housing improvements with physical health, PCS-12 SF-12v2 scale, over 65	β = −3.35 (−4.68, −2.02), p<0.05
			Association of kitchen and bathroom with physical health, PCS-12 SF-12v2 scale, over 65	β = −3.08 (−4.89, −1.27), p<0.001
			Association of central heating with physical health, PCS-12 SF-12v2 scale, over 65	β = −4.46 (−6.66, −2.26), p<0.05
			Association of doors with physical health, PCS-12 SF-12v2 scale, over 65	β = −4.30 (−6.32, −2.27), p<0.05
			Association of fabric works with physical health, PCS-12 SF-12v2 scale, over 65	β = −2.81 (−4.53, −1.09), p<0.05
			Association of housing improvements with mental health, MCS-12 SF-12v2 scale, over 65	β = 7.18 (5.94, 8.42), p<0.05
			Association of kitchen and bathroom with mental health, MCS-12 SF-12v2 scale, over 65	β = 8.19 (6.52, 9.85), p<0.05
			Association of central heating with mental health, MCS-12 SF-12v2 scale, over 65	β = 6.92 (4.65, 9.19), p<0.05
			Association of doors with mental health, MCS-12 SF-12v2 scale, over 65	β = 7.74 (5.66, 9.81), p<0.05
			Association of fabric works with mental health, MCS-12 SF-12v2 scale, over 65	β = 6.73 (5.14, 8.32), p<0.05
Edwards et. al. 2016	Structural	Quality of life	Household health status	MC = 3.25
			Main tenant health status	MC = 4.85
			Health-related quality of life	MC = -0.01
			Mental well-being	MC = 0.46
			Life satisfaction	MC = 0.09
			Happiness	MC = 0.25
			Anxiety	MC =-0.39
			Financial satisfaction	MC = 0.36
		Non-health outcomes	Rooms unheated	MC = -0.73
			GP visits in six months	Reduced by 10%

		Health service utilisation	Hospital visits in six months<br>Accident and emergency attendance in six months<br>Inpatient stays in six months	Reduced by 67%<br>Reduced by 45%<br>Reduced by 4%
		Cost analysis	Household access on NHS health services	£94 cost reduction per household, £45 per tenant
Fyfe 2021	Structural	Health service utilisation	Rate of prescriptions dispensed for infectious diseases	RR = 0.98 (0.98-0.99), p<0.05
			Rate of prescriptions dispensed for prevention of symptoms of chronic respiratory diseases	RR = 1.00 (0.99-1.00)
			Overall rate exacerbation sensitive medication of prescriptions dispensed to treat chronic respiratory disease, all	RR = 0.96 (0.96-0.97), p<0.05
			Rate exacerbation sensitive medication of prescriptions dispensed to treat chronic respiratory disease, 15-64	RR = 0.95 (0.94-0.96), p<0.05
			Rate exacerbation sensitive medication of prescriptions dispensed to treat chronic respiratory disease, 65 and over	RR = 0.97 (0.96-0.98), p<0.05
			Overall rate, prevention of symptoms of chronic disease incidence by medication measure	RR = 0.93 (0.88-0.98), p<0.05
			Prevention of symptoms of chronic disease incidence by medication measure, age 15-64	RR = 0.91 (0.85-98), p<0.05
			Prevention of symptoms of chronic disease incidence by medication measure, age 65 and over	RR = 0.92 (0.85-1.01)
			Prevention of symptoms of chronic disease incidence by medication measure, European ethnicity	RR = 0.91 (0.86- 0.97), p<0.05
			Prevention of symptoms of chronic disease incidence by medication measure, least deprived	RR = 0.85 (0.75-0.96), p<0.05
			Prevention of symptoms of chronic disease incidence by medication measure, whole house insulation	RR = 0.90 (0.84-0.85), p<0.05
			Overall hospital admission rates	RR = 0.89 (0.88-0.90), p<0.05
			Overall hospital admission rates, aged 15-64	RR = 0.89 (0.86-0.91), p<0.05
			Overall hospital admission rates, 65 and over	RR = 0.88 (0.86-0.94), p<0.05
			Hospital admissions per 1,000 people	RR = 9.26 (9.05-9.47), p<0.05

			Hospital admissions, asthma	RR = 0.80 (0.70-0.90), p<0.05
			Hospital admissions, COPD adults aged 65 and over	RR = 0.80 (0.70-0.91), p<0.05
			Hospital admissions, ischaemic heart disease adults aged 65 and over	RR = 0.75 (0.66-0.83), p<0.05
			Hospital admissions, whole house insulation	RR = 0.88 (0.85-0.90), p<0.05
			Hospital admissions, ceiling insulation	RR = 0.88 (0.84-0.92), p<0.05
			Hospital admissions, heating	RR = 0.89 (0.84-0.92)
		Mortality	No. of deaths, all cause mortality	N = 4,569 (1.23%)
			Cold-associated mortality	N = 1,916 (0.52%)
			No. of deaths of people 65 and over with an all-cause hospital admission at baseline and died at follow-up, all cause mortality	N = 1,516 (15.30%)
			No. of deaths of people 65 and over with an all-cause hospital admission at baseline and died at follow-up, cold-associated mortality	N = 776 (7.83%)
			No. of deaths of people 65 and over with a cold-associated hospital admission at baseline and died at follow-up, all cause mortality	N = 916 (19.20%)
			No. of deaths of people 65 and over with a cold-associated hospital admission at baseline and died at follow-up, cold-associated mortality	N = 558 (11.70%)
			Adjusted survival rates for all-cause mortality	HR = 1.01 (0.97-1.06)
			Adjusted survival rates for all-cause mortality, 15-64 years	HR = 1.11 (1.02-1.22), p <0.05
			Adjusted survival rates for all-cause mortality, 65 and over	HR = 0.98 (0.94-1.03)
			Unadjusted survival from cold-associated mortality	HR = 1.00 (0.93-1.06)
			Cold-associated mortality, all types of intervention, aged 65 and over cold-associated hospital admissions	HR = 1.44 (1.00-1.30)
			Cold-associated mortality, insulation & heater, aged 65 and over cold-associated hospital admissions	HR = 0.84 (0.53-1.36)

			Cold-associated mortality, ceiling & underfloor insulation, aged 65 and over cold-associated hospital admissions	HR = 1.15 (0.97-1.38)
			Cold-associated mortality, ceiling insulation only, aged 65 and over cold-associated hospital admissions	HR = 1.28 (0.99-1.67)
			Cold-associated mortality, underfloor insulation only, aged 65 and over cold-associated hospital admissions	HR = 1.10 (0.74-1.65)
			Cold-associated mortality, insulation top-up, aged 65 and over cold-associated hospital admissions	HR = 0.58 (0.21-1.59)
Heyman et al. 2011	Structural	Physical health	Overall reported health status	Not reported
			Scores on a symptom check list	Not reported
			Improvements in specific conditions	Not reported
		Health service utilisation	Frequency of health service usage	Not reported
		Quality of life	SF36 overall or subscale scores, quality of life	Not reported
			Social functioning subscale of the SF36	r=.23, N=83, p.=.03
		Non-health outcomes	Fuel efficiency, SAP rating	Mean = 56.1 (11.6)
			Room temperatures, living room PM	t = 2.19, p=0.03
			Estimated fuel expenditure	z = 0.827
			Difference from required fuel expenditure	z = 2.031
			Correlation between year 3 SAP ratings, fuel expenditure, and room temperatures	r=0.37, N=94, P<.0001
			Satisfaction with home warmth, years 2 and 3	Wilcoxon W=6147, N=172, P=.02
Liddell et al 2011	Structural	Mortality	Estimated deaths prevented by all home safety checks (fire safety, smoke detector, carbon monoxide)	0.81
			Estimated injuries prevented by all home safety checks (fire safety, smoke detector, carbon monoxide)	22.11
		Cost analysis	Estimated savings, improved impact on mental well-being (Loft insulation)	Reduced by 20% = 1,811 fewer, 1 QALY per 238 people = 7.61 QALY, Estimated saving £1,141,500

			Estimated savings, improved impact on mental well-being (Cavity wall insulation)	Reduced by 40% = 1,808 fewer, 1 QALY per 238 people = 7.60 QALY, Estimated saving £1,140,000
			Estimated savings, physical injury and deaths on ALL home safety checks	£1,150,200 estimated saving on fatalities prevented, £150,300 estimated savings on injuries prevented
			Improved mental well-being and physical health, central heating via local funding	Estimated savings of £758,520
			Improved mental well-being and physical health, central heating via local Warm Front Funding	Estimated savings of £512,820
			Total Mental well-being, physical health, and lives saved	Estimated savings of £4,853,340
Osman et al 2010	Structural	Non-health outcomes	National Home Energy Rating (NHER)	β = 0.2 (-0.1–0.6)
			Estimated Annual Fuel Costs (EAFC)	β = -12.1 (-52.4–28.7)
			Living room 21c	β = 7.4 (-11.0–25.8)
			Bedroom 18c	β = 22.4 (1.6–43.4)
			Living room average humidity	β = -1.7 (-4.9–1.6)
			Bedroom average humidity	β = -0.8 (-3.5–1.9)
		Health service utilisation	COPD admissions N	β = 0.4 (-0.4–1.1)
		Quality of life	Symptom score from SGRQ	β = -3.5 (-11.3–4.3)
			Impact score from SGRQ	β = -3.0 (-4.3–10.2)
			Activities score from SGRQ	β = -1.4 (-7.7–4.8)
			SGRQ total score	β = -0.9 (-6.7–4.9)
			VAS Score	β = -0.3 (-1.2–0.6)
Page et al 2022	Structural	Quality of life	SF-36: Mental component Summary (MCS)	β = 1.73 (0.21-3.25), p=0.026
			SF-36: Physical component Summary (PCS)	β = 0.81 (-0.30–1.92)
			EQ-5D-5L	β = 0.01 (-0.03–0.04)
			Adult Social Care Outcomes Toolkit (ASCOT)	β = 0.024 (0.006–0.042), p=0.009
		Cost analysis	QALY gain	t = 0.45, mean QALY gain is 0.01 (0.08)
			Medicare Benefits Scheme (MBS) services used over winter	β = -0.10 (-0.21–0.01)

		Health service utilisation	GP services used over winter	β = 0.02 (0.11–0.14)
			MBS charges over winter	β = -157 (-311–2), p=0.046
			MBS benefits paid over winter	β = -108 (-230-14)
			MBS out-of-pocket costs	β = -49 (-98-0), p=0.051
			Pharmaceutical Benefits Scheme services, all medicines available to be dispensed to patients at a government-subsidised price	β = -0.02 (-0.06-0.09)
			Number of hospitalisations over winter	β = -0.17 (-0.57-0.23)
			Hospital admission costs over winter	β = -557 (-1417-302)
			Hospital length of stay over winter	β = 0.12 (-0.33-0.58)
			Number of ED visits over winter	β = 0.03 (-0.31-0.36)
			Number of healthcare services used over winter	β = -1.29 (-3.67-1.08)
		Mortality	Number of deaths over winter	β = -0.87 (-1.97-0.23)
		Cost analysis	Total healthcare costs over winter	β = £ -887 (-106-879), p=0.08
			Total VHHP Costs (Costs-Cost savings)	$4684
			Cost effectiveness analysis, mean temperature and costs	Mean effect = 0.32, p = 0.026; Cost = $4810
			Incremental cost-effectiveness ratio (ICER)	$15,232
			Cost benefit analysis, cost savings	Year 7 net costs = - $-271
		Non-health outcomes	Winter temperature, indoor	β = 0.33 (0.05-0.60), p=0.022
			Morning temperature, indoor	β = 0.47 (0.10-0.84), p=0.012
			Cold exposure	β = -43 (-88-2), p=0.012
			Subjective thermal comfort	β = 2.3 (1.8-3.0), p<0.001
			Mould reported in households	OR = 1.07 (0.79-1.45)
			Damp or musty smell	OR = 1.37 (0.99-1.89), p=0.061
			Condensation	OR = 1.48 (1.12-1.95), p=0.006
Peralta et al. 2017	Structural	Mortality	Associations between extreme cold temperatures and mortality from natural causes, all	RR = 1.95 (1.15-3.33) Lag 15-17
			Associations between extreme cold temperatures and mortality from natural causes, men	RR = 2.23 (1.14-4.36) Lag 15-17
			Associations between extreme cold temperatures and mortality from natural causes, women	RR = 0.46 (0.21-1.01) Lag 0-2

			Associations between extreme cold temperatures and mortality from respiratory and circulatory diseases, men	RR = 2.80 (1.23-6.34) Lag 15-17
			Associations between extreme cold temperatures and mortality from respiratory and circulatory diseases, women	RR = 0.27 (0.11-0.67) Lag 0-2
			Associations between extreme cold and mortality, circulatory diseases, men	RR = 3.02 (0.70-12.98)
			Associations between extreme cold and mortality, respiratory diseases, men	RR = 6.01 (1.10-32.85) Lag 15-17
			Associations between extreme cold and mortality, men aged 75+	RR = 4.18 (1.22-14.31) Lag 15-17
			Associations between extreme cold and mortality, circulatory diseases, women	RR = 0.10 (0.03-0.39) Lag 0-2
			Associations between extreme cold and mortality, respiratory diseases, women	RR = 0.78 (0.03-19.52) Lag 0-2
			Associations between extreme cold and mortality, women aged 75+	RR = 0.53 (0.20-1.39) Lag 0-2
Pollard et al. 2019	Behavioural	Quality of life	Changes in physical health and well-being	Not reported, p>0.05
		Health service utilisation	Use of GP for any respiratory, cardiovascular, circulatory, mental health conditions, or cold/flue	Not reported, p=0.016
			Use of hospital for any respiratory, cardiovascular, circulatory, mental health conditions, or cold/flue	Not reported
			Use of A&E for any respiratory, cardiovascular, circulatory, mental health conditions, or cold/flue	Not reported, p<0.001
			Use of NHS services, such as pharmacist, medicines, GP home visits, etc.	Not reported, p=0.001
		Mental health	Wellbeing, awareness of temperature of the house	Not reported, p=0.016
			Wellbeing, feeling vulnerable during winter	Not reported, p=0.071
			Wellbeing, feeling cold during winter	Not reported
			Wellbeing, feeling poorly during winter compared to last year	Not reported
Poortinga et al. 2017	Structural	Non-health outcomes	Associations of the intervention measures with the housing suitability	OR = 1.14 (1.09-1.19)

			Associations of the intervention measures with satisfaction in property's state of repair	β = 0.104 (0.008), p<0.001
			Associations of the intervention measures with reporting any building problems	OR = 0.90 (0.87-0.93)
			Associations of total spend and housing suitability	OR = 1.03 (1.02-1.05)
			Associations of total spend with satisfaction in property's state of repair	β = 0.028 (0.002), p<0.001
			Associations of total spend with reporting any building problems	OR = 0.97 (0.96-0.98)
			Associations of the intervention measures with thermal comfort and household finances	Thermal comfort β = −0.015 (0.033); Costs of living in home β = 0.025 (0.033); Difficulties in paying bills β = 0.007 (0.066)
			Associations of each additional intervention measure with the thermal comfort	β = 0.090 (0.008), β = 0.024 (0.002), p<0.001
			Associations of each additional intervention measure with better household finances	Costs of living in home β = -0.072 (0.008), p<0.001; Difficulties in paying bills OR = 0.96 (0.93-0.99)
			Associations of total spend with better household finances	Costs of living in home β = -0.017 (0.002), p<0.001; Difficulties in paying bills OR = 0.99 (0.98-1.00)
		Physical health	Association of cavity wall insulation with general health	OR = 0.78 (0.68-0.89)
			Association of cavity wall insulation with respiratory symptoms	OR = 1.47 (1.30-1.66)
			Association of windows and doors with good health	β = -0.15 (0.099)
			Association of boilers with respiratory symptoms	β = -0.008 (0.057)
			Association of total spend with respiratory symptoms	OR = 0.99 (0.98-1.00)
			Association of each additional intervention measure with physical health	OR = 1.03 (1.00-1.07)
			Association of total spend with physical health	OR = 1.01 (1.00-1.02)
		Mental health	Association of cavity wall insulation with mental health	β = -2.052 (0.687), p<0.05
			Association of each additional intervention measure with mental health	β = 0.0826 (0.161), p<0.001
			Association of total spend with mental health	β = 0.223 (0.044), p<0.001

Poortinga et al. 2018a	Structural	Health service utilisation	Change in cardiorespiratory emergency admissions for people of all ages	Δ= 0.0011 (–0.0103-0.0125)
			Change in COPD emergency admissions for people of all ages	Δ= -0.0002 (–0.0025-0.0022)
			Change in cardiovascular-related emergency admissions for people of all ages	Δ= -0.0014 (–0.0083-0.0055)
			Change in respiratory-related admissions for people of all ages	Δ= 0.0042 (–0.0046-0.0131)
			Change in cardiorespiratory emergency admissions for people aged > 60	Δ= 0.0490 (–0.0153-0.1132)
			Change in COPD emergency admissions for people aged > 60	Δ= 0.0133 (–0.0030-0.0295)
			Change in cardiovascular-related emergency admissions for people aged > 60	Δ= 0.0273 (0.0068-0.0479), p=0.009
			Change in respiratory-related admissions for people aged > 60	Δ= 0.0412 (–0.0141-0.0964)
Poortinga et al. 2018b	Structural	Physical health	Self-reported respiratory symptoms	β = -0.141 (0.202)
			Self-reported asthma symptoms	β = -0.133 (0.253)
		Quality of life	Mental health composite score of the SF-12 (MCS)	β = -0.003 (0.812)
			Physical health composite score of the SF-12 (PCS)	β = 0.976 (0.669)
			Health-related quality of life SF-6D and QALY	β = –0.007, SE (0.016) (–0.04-0.02)
		Mental health	Subjective wellbeing	β = 0.384 (0.134)
		Non-health outcomes	Putting up with feeling cold to save costs	OR = 0.49(0.25-0.94)
			Financial difficulties	β = -0.15 (-0.25--0.05)
			Thermal satisfaction	β = 1.34 (0.91-1.77)
			Satisfaction of the state of repair of the home	β = 1.35 (0.92-1.79)
			The number of housing problems	β = -1.10 (-1.54-0.66)
			Social interactions	OR = 0.32 (0.13-0.77)
Poortinga et al. 2018c	Structural	Cost analysis	Costs and consequences for hospital admissions for cardiovascular and respiratory conditions	0.0041 events per person, per-person cost £345.75, intervention cost £334.44, increase in treatment costs of £11.31
			Costs and consequences for hospital admissions for COPD	–0.0006 events reduction per person, reduction in NHS treatment costs of £1.64

			Costs and consequences for hospital admissions for cardiovascular-related	–0.0053 events per person, discounted amount saved is £14.56
			Costs and consequences for hospital admissions for respiratory-related	0.016 events per person, net cost £248.48
			Within-year QALY decrease for a single COPD exacerbation hospital admission	0.022, ICER is > £10M (£10,485,472.12) per QALY gained
Preval et al 2017	Structural	Health service utilisation	Health risk, insulation intervention for circulatory-related hospital admissions	HR = 0.673 (0.535-0.847), p=0.001
			Health risk, insulation intervention for respiratory-related hospital admissions	HR = 0.83 (0.655-1.051)
			Health risk, heating intervention for circulatory-related hospital admissions	HR = 1.348 (0.881-2.064)
			Health risk, heating intervention for respiratory-related hospital admissions	HR = 1.129 (0.656-1.944)
			Health risk, insulation and heating intervention for circulatory-related hospital admissions	HR = 0.581 (0.350-0.964)
			Health risk, insulation and heating intervention for respiratory-related hospital admissions	HR = 0.624 (0.324-1.200)
Rodgers et al 2018	Structural	Health service utilisation	Windows and doors, emergency hospital admissions, 60 years and over, exposure 1	IRR = 0.71 (0.63-0.81), p=0.000
			Wall insulation, emergency hospital admissions, 60 years and over, exposure 1	IRR = 0.75 (0.67-0.84), p=0.000
			Loft insulation, emergency hospital admissions, 60 years and over, exposure 1	IRR = 0.98 (0.86-1.11)
			Heating systems, emergency hospital admissions, 60 years and over, exposure 1	IRR = 0.91 (0.82-1.01)
			Kitchens, emergency hospital admissions, 60 years and over, exposure 1	IRR = 0.98 (0.83-1.17)
			Bathrooms, emergency hospital admissions, 60 years and over, exposure 1	IRR = 0.93 (0.81-1.06)
			Electrical systems, emergency hospital admissions, 60 years and over, exposure 1	IRR = 0.61 (0.53-0.72), p=0.000
			Garden paths, emergency hospital admissions, 60 years and over, exposure 1	IRR = 0.61 (0.64-0.83), p=0.000

			Windows and doors, cardiovascular emergency hospital admissions, 60 years and over, exposure 1	IRR = 0.81 (0.69-0.96)	
			Wall insulation, cardiovascular emergency hospital admissions, 60 years and over, exposure 1	IRR = 0.73 (0.63-0.85), p=0.000	
			Loft insulation, cardiovascular emergency hospital admissions, 60 years and over, exposure 1	IRR = 0.86 (0.73-1.02), p=0.0835	
			Heating systems, cardiovascular emergency hospital admissions, 60 years and over, exposure 1	IRR = 0.94 (0.82-1.08)	
			Kitchens, cardiovascular emergency hospital admissions, 60 years and over, exposure 1	IRR = 0.91 (0.73-1.13)	
			Bathrooms, cardiovascular emergency hospital admissions, 60 years and over, exposure 1	IRR = 0.94 (0.78-1.13)	
			Electrical systems, cardiovascular emergency hospital admissions, 60 years and over, exposure 1	IRR = 0.80 (0.66-0.99), p=0.0364	
			Garden paths, cardiovascular emergency hospital admissions, 60 years and over, exposure 1	IRR = 0.84 (0.70-1.00)	
			Windows and doors, respiratory emergency hospital admissions, 60 years and over, exposure 1	IRR = 0.61 (0.49-0.76), p=0.000	
			Wall insulation, respiratory emergency hospital admissions, 60 years and over, exposure 1	IRR = 0.76 (0.62-0.92), p=0.0055	
			Loft insulation, respiratory emergency hospital admissions, 60 years and over, exposure 1	IRR = 1.18 (0.95-1.48)	
			Heating systems, respiratory emergency hospital admissions, 60 years and over, exposure 1	IRR = 0.85 (0.71-1.03)	
			Kitchens, respiratory emergency hospital admissions, 60 years and over, exposure 1	IRR = 1.17 (0.86-1.59)	
			Bathrooms, respiratory emergency hospital admissions, 60 years and over, exposure 1	IRR = 0.89 (0.70-1.13)	
			Electrical systems, respiratory emergency hospital admissions, 60 years and over, exposure 1	IRR = 0.43 (0.33-0.57, p=0.000	
			Garden paths, respiratory emergency hospital admissions, 60 years and over, exposure 1	IRR = 0.62 (0.49-0.78), p=0.000	
Saeki et al. 2015	Behavioural	Physical health	Sleep-trough morning BP surge, SBP (mmHg)<br>Prewaking MBPS, DBP (mmHg	β = -4.43 (-7.88--0.97), p=0.012<br>β = -2.33 (-0.08--4.58), p=0.042	

			Physical activity (counts/min)	β = -26.10 (-55.52-3.30), p=0.082
		Non-health outcomes	Living room temperature (C)	β = 2.09 (1.28-2.90), p<0.001
Telfar-Barnard et al 2011	Structural	Mortality	Effect of treatment on mortality rates in people aged 65+ hospitalised prior to treatment month	RR = 0.95,(0.80-1.13)
			Effect of treatment on mortality rates in people aged 65+ hospitalised with circulatory illness prior to treatment month	RR = 0.73, (0.53-1.00), p=0.048
			Effect of treatment on mortality rates in people aged 65+ hospitalised with respiratory illness prior to treatment month	RR = 1.01 (0.73-1.40)
		Cost analysis	The value of reduced mortality per dwelling, life years gained	$439.95 ($0.00, $765.84)
			The value of reduced mortality per dwelling, CSC and non-CSC	CSC dwelling = $ 613.05 ($0.00, $1,067.16); Non CSC dwelling = $216.38 $0.00, $376.66)
			Change in monthly hospitalisation costs, insulation	β = -$5.37 (-9.54, -1.21), p<0.01
			Change in monthly hospitalisation costs, heating	β = -$1.37 (-10.05, 7.32)
			Change in monthly pharmaceutical costs, insulation	β = -$0.92 (-1.57, -0.27), p<0.01
			Change in monthly pharmaceutical costs, heating	β = -$0.68 (-2.03, 0.66)
			On-going annual benefit for retrofitted insulation	$563.18 ($121.23-$889.07)
			On-going annual benefit for heating	$4.64
Tonn et al 2023	Structural	Mental health	Thinking about your mental health, which includes stress, depression, and problems with emotions, for how many days during the past 30 days was your mental health not good?	β = 1.34
		Physical health	In the past three months, have you had headaches that are either new or more frequent or severe than ones you've had before? Y/N	β = -6.4, p<0.001
			How many days per week do you do moderate physical activities for at least 30 min?	β = 0.15
			How many days per week do you do vigorous physical activities for at least 20 min?	β = 0.01
			Had arthritis symptoms – past 3 months	β = -0.09, p<0.10

		Health service utilisation	Visited doctor for arthritis last 12 months	β = -0.094
			Stay overnight in the hospital because apartment was too cold	β = -0.006
			Go to emergency at hospital because apartment was too cold	β = -0.016, $p<0.05$
			Visit a doctor because apartment was too cold	β = -0.031, $p<0.01$
			Urgent care for treatment of worsening arthritis symptoms - last 12 months	β = -0.024
			Visited emergency department because of arthritis – last 12 months	β = -0.043
			Stay overnight in hospital because of arthritis – last 12 months	β = -0.026
			Other than a routine visit, have you had to see a doctor in the past 12 months for symptoms related to shortness of breath, bronchitis, or other COPD, or emphysema flare ups?	β = 0.326, $p<0.05$
			Did you have to visit an emergency room or be admitted to a hospital in the past 12 months because of your COPD, chronic bronchitis, or emphysema?	β = -0.049
			How many different medications do you currently take each day to help with your COPD, chronic bronchitis, or emphysema?	β = -0.042
